# Metallothionein from Wild Populations of the African Catfish *Clarias gariepinus*: From Sequence, Protein Expression and Metal Binding Properties to Transcriptional Biomarker of Metal Pollution

**DOI:** 10.3390/ijms18071548

**Published:** 2017-07-18

**Authors:** Ethel M’kandawire, Agnieszka Mierek-Adamska, Stephen R. Stürzenbaum, Kennedy Choongo, John Yabe, Maxwell Mwase, Ngonda Saasa, Claudia A. Blindauer

**Affiliations:** 1 Department of Chemistry, University of Warwick, Coventry CV4 7AL, UK; ethel.mkandawire@unza.zm (E.M.); mierek_adamska@umk.pl (A.M.-A.); 2 School of Veterinary Medicine, University of Zambia, P.O. Box 32379, Lusaka 10101, Zambia; k.choongo@unza.zm (K.C.); mjyabe@yahoo.co.uk (J.Y.); mmwase@yahoo.com (M.M.); nsaasa@gmail.com (N.S.); 3 Department of Genetics, Faculty of Biology and Environmental Protection, Nicolaus Copernicus University, 87-100 Torun, Poland; 4 Analytical and Environmental Sciences Division, Faculty of Life Sciences & Medicine, King’s College London, London SE1 9NH, UK; stephen.sturzenbaum@kcl.ac.uk

**Keywords:** Kafue River, heavy metals, *Clarias gariepinus*, liver, metallothionein, MT gene expression, biomarker, toxicogenomics

## Abstract

Anthropogenic pollution with heavy metals is an on-going concern throughout the world, and methods to monitor release and impact of heavy metals are of high importance. With a view to probe its suitability as molecular biomarker of metal pollution, this study has determined a coding sequence for metallothionein of the African sharptooth catfish *Clarias gariepinus*. The gene product was recombinantly expressed in *Escherichia coli* in presence of Zn(II), Cd(II), or Cu, and characterised by Electrospray Ionisation Mass Spectrometry and elemental analysis. *C. gariepinus* MT displays typical features of fish MTs, including 20 conserved cysteines, and seven bound divalent cations (Zn(II) or Cd(II)) when saturated. Livers from wild *C. gariepinus* fish collected in all three seasons from four different sites on the Kafue River of Zambia were analysed for their metal contents and for MT expression levels by quantitative PCR. Significant correlations were found between Zn and Cu levels and MT expression in livers, with MT expression clearly highest at the most polluted site, Chililabombwe, which is situated in the Copperbelt region. Based on our findings, hepatic expression of MT from *C. gariepinus* may be further developed as a major molecular biomarker of heavy metal pollution resulting from mining activities in this region.

## 1. Introduction

Heavy metal contamination of fresh water systems is of serious concern worldwide because metals have the ability to bioaccumulate in aquatic organisms, and, in contrast to organic pollutants, are non-degradable [1,2,3]. Some metals such as Fe, Cu, Co, Mn and Zn are essential to biological life forms, but become toxic above their threshold concentrations [4,5]. Other metals including As, Cd, Hg and Pb are non-essential and may be toxic even at minute concentrations [6]. Heavy metals enter aquatic systems by either natural or anthropogenic sources [6]. Metals released into aquatic bodies may be dissolved or suspended in water, deposited in sediments, or assimilated in aquatic biota [7]. In fish, heavy metals may cause physiological, biochemical, morphological and haematological changes as well as oxidative stress [8]. Because fish is a major component of the human diet in many parts of the world, bioaccumulation of heavy metals in commercial edible fish may pose health risks to fish consumers [9]. Acute heavy metal toxicity in man causes amongst other symptoms tremor, ataxia, paralysis, convulsions, haemoglobinuria, and gastrointestinal disorders such as diarrhoea, vomiting and stomatitis [6,10]. Chronic exposure may lead to neurotoxic, carcinogenic, mutagenic or teratogenic effects [10]. A prominent example of river water-derived heavy metal poisoning is that of Itai-Itai (Ouch-Ouch) disease, a combination of osteomalacia, osteoporosis and pain in bones, which was discovered in the 1950s and was caused by cadmium contamination of the Jinzu River [11].

In Zambia, the Kafue River has been receiving effluents from industrial, agricultural, mining and domestic/sewage wastes over a long period of time, leading to the deterioration of the river’s ecosystems. In particular, mining waste from the Copperbelt mining area has been implicated in environmental degradation of Kafue River ecosystems [12,13]. To begin to mitigate the effects of mining activities, heavy metal concentrations in the Kafue River have been measured in water [14], sediments [15,16] and biota [16,17]. However, studies on monitoring the extent and effects of metal pollution in the river through biomarker studies are extremely scarce [14]. A biomarker is defined as any biological response measured inside an organism at molecular, biochemical or cellular level or in an organism’s products (urine, faeces, feathers and others) indicating a departure from the normal status as a result of exposure to potentially toxic chemicals [18]. The most compelling reason for using biomarkers is that they can give information on the biological effects of pollutants rather than a mere quantification of their environmental levels. In the case of heavy metals, biomarker monitoring involves the assessment of exposure and effects of heavy metals in organisms by determining early adverse alterations that may be partly or fully reversible, and by examining the occurrence of irreversible diseases or tissue damage in organisms [1,3].

The potential of metallothioneins (MTs) to be used to measure an organism’s exposure and response to toxic levels of metal ions was recognised soon after their discovery [19]. Meanwhile, MTs have been proposed for or already have been used in biomonitoring programmes to characterise metal contamination in aquatic environment [20,21,22,23,24,25,26,27,28]. MTs occur in virtually all eukaryotes and some bacteria [29]. They constitute a super-family of soluble, cysteine-rich, low molecular weight (usually <10 kDa) proteins with high affinity and capacity to bind thiophilic metal ions (in particular Zn(II), Cu(I), Cd(II), Hg(II) and Ag(I)) in characteristic metal-thiolate clusters [29,30,31,32,33]. The MT super-family is unusual in that it is polyphyletic, i.e., its members are thought to originate not from a common ancestor, but to be examples of convergent evolution [34]. Thus, since this super-family is defined mainly through chemical properties rather than structure and function, its members have several different biological functions, and the main function may differ depending on organism and tissue [35]. In vertebrates including fish, the primary function of MTs concerns homeostasis of essential metals such as Cu and Zn, keeping these metal ions safe but potentially available within the intracellular environment [35,36]. In addition, their high thiol content renders MTs redox-active [37] and able to respond to oxidative stress [35,38]. Finally, owing to their inducibility by a range of heavy metals [30] and enhanced tolerance towards acute administration of these [39,40], their role in detoxifying non-essential, toxic metals is widely cited. Exposure to excessive levels of essential metals (e.g., Zn and Cu) or toxic metals (e.g., Cd, Hg, Ni, Ag, Au, Pb, As, and Bi) leads to at least temporary increases of these metals in the cytosol and nuclei of cells [41]. One response of the cell to this situation consists in the upregulation of MT gene transcription [30]; this is at least in part mediated through the action of metal-responsive elements (MREs) found in the promoter regions of MT genes [42], including those of fish [43,44]. Like those of mammals [45], fish MT gene promoter regions also contain response elements for oxidative stress (antioxidant response elements (AREs) and activator protein-1 (AP-1) binding sites), glucocorticoids (GREs) and cytokines [46,47]; thus, heavy metals are not the only factors affecting MT transcription. Furthermore, the actual molecular mechanisms for MT induction by xenobiotic heavy metals are still not entirely clear; an initial hypothesis involving Zn(II) displacement from basal MT by the incoming metal, followed by activation of the MRE-binding zinc sensor MTF-1 is not entirely consistent with experimental evidence [48,49].

Irrespective of unclear mechanistic details, increased expression of MTs in response to harmful levels of metals has been demonstrated in fish [3,26,44,50]. The capacity for MT induction is greatest in tissues that are active in uptake, storage and excretion, such as the small intestine, liver and gills [2,20,26,51]. Although not all fish species respond to heavy metal exposure by MT induction [52], numerous studies have concluded that MTs are sensitive and efficient biomarkers for evaluating the cumulative biological effects of metal exposure in fish [3,26,27,28]. MT induction can in principle be measured either at protein [51,53,54,55] or mRNA level. Quantifying protein, especially when combined with chromatography and elemental analysis, allows determination of which metal ions the MT is associated with [56,57]. However, for biomarker studies, mRNA may not only be easier to measure, but also more reliable, as mRNA does not necessarily become translated, and/or protein levels do not always reflect the degree of exposure [23,58]. An extreme case concerns a hemoglobinless ice fish, in which massive increases in hepatic mRNA were accompanied by undetectable MT protein [59]. For these reasons, mRNA quantitation may provide a more sensitive measure of heavy-metal exposure.

Several laboratory studies [24,60], as well as a number of field studies in natural fish populations [23,55,61] around the globe have successfully employed fish MT mRNA quantification as a biomarker of heavy metal stress (also see [3,26] and references therein). However, although a number of studies have investigated heavy metal accumulation in sediments and fish in Zambia [16,62], MT expression in Kafue River fish, and any correlation with pollution-related heavy metal bioaccumulation has not been explored so far.

Previously, one of the authors of the present study has demonstrated a positive correlation between MT expression and liver metal contents in the antelope Kafue lechwe (*Kobus leche kafuensis*) [63]. For the present study, the African sharptooth catfish (*Clarias gariepinus*) was selected as model species. *C. gariepinus* is found in unpolluted and polluted aquatic environments throughout many African and Asian countries. It grows at a fast rate and can live up to eight years, and thus has potential to accumulate high levels of heavy metals. Catfish are also omnivorous [64] and bottom feeders (benthic species) [65], and are thus readily exposed to metals that accumulate in river sediments. Hence, they are particularly useful as bio-indicators of aquatic pollution. Moreover, *C. gariepinus* is a popular delicacy relished throughout tropical Africa and if heavily contaminated, may pose health risks to its consumers [66]. Metal bioaccumulation and biomarkers of heavy metal pollution have been studied previously in *C. gariepinus* [64,67,68].

Metal-induced MT induction in liver, as the main detoxification organ, is well-documented, including in fish in laboratory culture [60,69,70] and in field studies involving feral fish [71,72,73,74]. Therefore, the current study determined heavy metal concentrations and MT expression levels in *C. gariepinus* livers at four sites along the Kafue River over three seasons. Since the genome of *C. gariepinus* has not yet been sequenced, cDNAs were amplified from MT mRNAs by reverse transcription polymerase chain reaction (RT-PCR) [75], and the sequences were determined. To check whether the cloned cDNA encodes functional protein, we also cloned the putative coding sequence into *E. coli* for overexpression. This allowed studies on metal binding properties of *C. gariepinus* MT protein. We found that *C. gariepinus* possesses at least one MT gene and that the translated protein sequence displays all typical hallmarks of fish MTs [36]. Like all other vertebrate MTs, the expressed protein bound seven molar equivalents of Zn(II) or Cd(II), whilst repeated attempts to isolate Cu-bound MT failed. Nonetheless, MT expression in 155 fish livers correlated most strongly with liver contents of Cu, besides those for Zn. We conclude that MT expression in *C. gariepinus* responds to elevated pollution with Cu resulting from mining activities in the Copperbelt region, and that MT expression may serve as a suitable biomarker of such pollution.

## 2. Results and Discussion

To evaluate hepatic MT as a biomarker of heavy metal pollution in *C. gariepinus* from the Kafue River, a cross-sectional study involving three time points and several study sites was conducted.

### 2.1. Study Area and Sampling Regime

**Figure 1:**
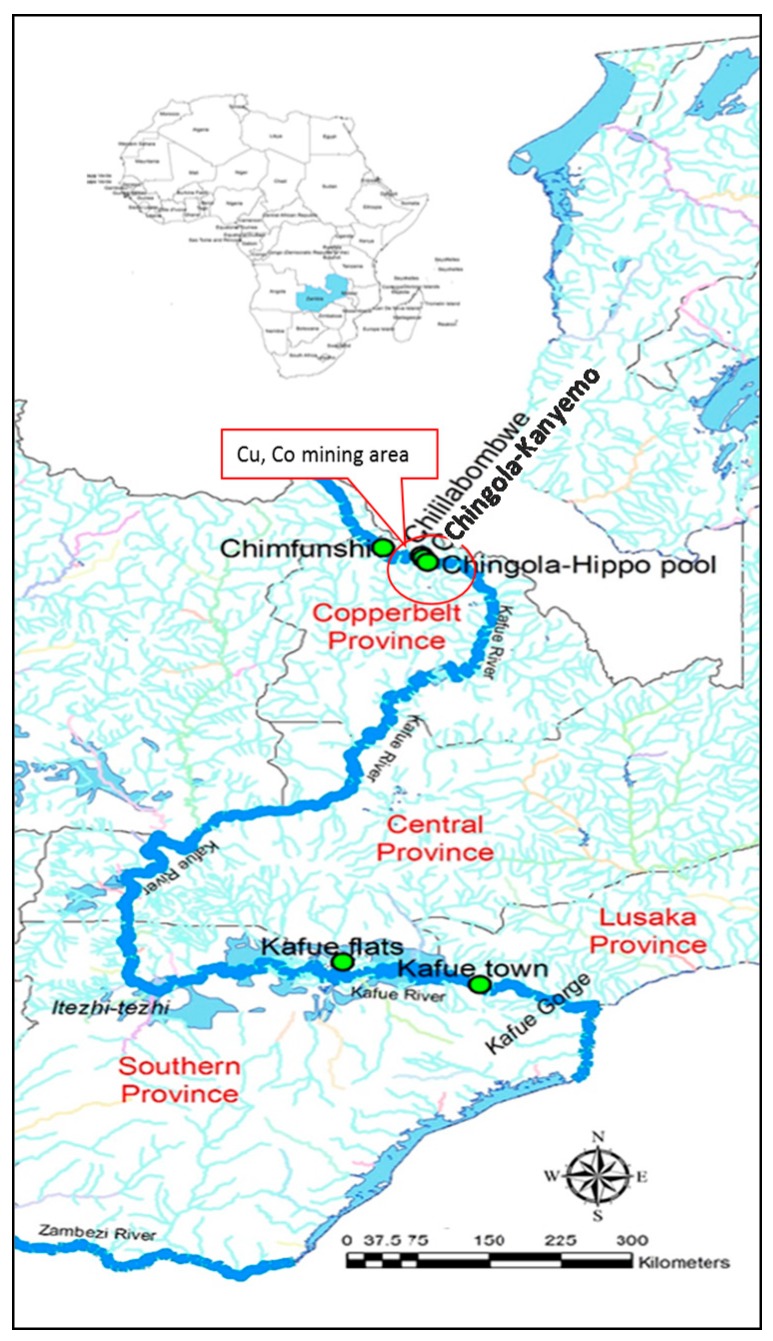
Map of Zambia showing the location of the Copperbelt mining area and sampling sites along the Kafue River.

The Kafue River is about 1600 km long; it originates in the North Western Province of Zambia and flows through four provinces, namely Copperbelt, Central, Southern and Lusaka provinces, before its confluence with the Zambezi River [76]. Over ten endemic species of fish including *C. gariepinus* are found in its waters [77]. Communities rely on these fish stocks for income and over 70% of their daily protein intake [78]. For our cross-sectional study, we initially selected six sites (Figure 1) along the Kafue River, namely Chimfunshi (non-industrialised area, upstream of the Copperbelt mining and industrial area, reference site), Chililabombwe, Chingola-Kanyemo and Chingola-Hippo Pool (all three sites within the Copperbelt mining and industrial area), Kafue Flats and Kafue Town (downstream of the Copperbelt mining and industrial area). The sites were purposively selected based on their proximity to the Copperbelt mining area, accessibility and presence of fishing camps. Fish and sediment samples were collected during 2014 for each of three seasons: warm-rainy (April), dry-cold (June) and dry-hot (September) season.

*C. gariepinus* fish were only obtained from Chimfunshi, Chililabombwe, Kafue Flats and Kafue Town; no fish were caught at Chingola-Kanyemo and Chingola-Hippo Pool in any season, probably because of high levels of heavy metal pollution resulting from mining waste discharge at these sites. Although factors relating to fishing patterns could have contributed to lack of fish, mining activities can reduce biodiversity and alter species composition in aquatic bodies [79]. Indeed, the disappearance of hippopotami (*Hippopotamus amphibius*) from the Kafue River in Chingola has been attributed to heavy metal accumulation from the Copperbelt mining area [80]. Our previous results on heavy metal levels in sediments of the Kafue River showed that the two Chingola sites were highly polluted throughout the year [81], although it is noteworthy that this was also true of Chililabombwe. The latter site is the most upstream of the three sites.

### 2.2. Identification of C. gariepinus Metallothionein cDNA Sequence

**Figure 2:**
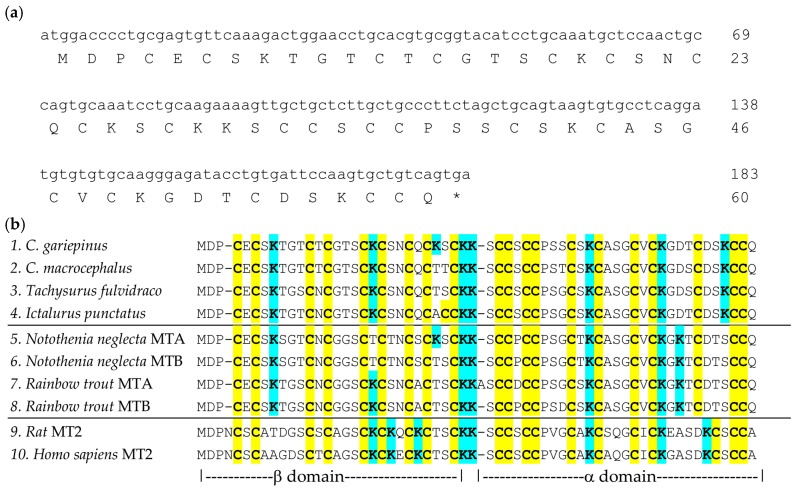
(**a**) Cloned cDNA sequence of *cgMT* and predicted amino acid sequence of CgMT. At the end of each line, the position of the last nucleotide/amino acid is marked; (**b**) Sequence alignment of CgMT (row 1) with MTs from other catfish (rows 2–4), other fish (rows 5–8), and two mammalian MTs (rows 9 and 10). Cysteines and lysines are highlighted in yellow and cyan, respectively.

The most essential prerequisite for biochemical biomarker studies, irrespective of whether these are based on the quantitation of mRNA or protein, is sequence information on the prospective biomarker. In the absence of a sequenced genome for *C. gariepinus*, MT coding regions were amplified from mRNA extracted from eight different livers from *C. gariepinus* collected at the four different sites. This required the design of degenerate primers; these were based on gene sequences of MTs from closely related fish species retrieved from GenBank (see Materials and Methods for details). PCR using these primers led to the clean production of PCR products of the expected size (Figure S1). Sequencing of these products followed by in silico analysis of the results revealed in each case the presence of a 183 bp coding sequence (CDS). The translation products of these sequences are proteins that contain 20 cysteine residues out of 60 amino acids (Figure 2a). We observed some small variations in some non-cysteine residues; these are suggested to be due to single-nucleotide polymorphism (see Figure S2 for respective cDNA sequences and translation products). The cDNA sequence (*cgMT* hereafter, accession no. KU999947) found in the majority of the eight fish was selected for further studies. This is the first report on a complete CDS for *C. gariepinus* MT; a partial CDS had been previously deposited at NCBI (accession no. DQ885944.1); over the 102 common nucleotide residues, our *cgMT* sequence shows 100% identity with DQ885944.1. Further BLASTN hits included the MTs from the closely related *C. macrocephalus* (AGC79139.1; 95% identity), and from *Poecilia reticulata* (XM_008412689.1; 79% identity). A BLASTP search with the predicted amino acid sequence of CgMT yielded also the MT from *C. macrocephalus* (AGC79139.1; 93% identity) and the channel catfish *Ictalurus punctatus* (NP_001187006.1; 93% identity).

The existence of MT-like proteins in fish has been recognised for some while [82]; there is now also a large body of work on the nucleotide level [36]. Some fish of the Salmoniformes family and Notothenoids have two MT genes [36] (also see Figure 2b for respective proteins), but most likely *C. gariepinus* only possesses a single MT gene, like *Ictalurus punctatus*, for which a sequenced genome is available [83]. The CgMT protein (Figure 2b) displays features typical of vertebrate MTs in general [29,30,35], and fish MTs in particular [36,84]. Its predicted molecular weight (excluding metal ions) is 6138.16 Da, typically low as for the vast majority of MTs. Its sequence is 60 amino acids long, with 20 Cys residues arranged in CC, CxC, and CxxC motifs, it is devoid of histidine or other aromatic residues, and it has a high proportion of lysine residues, i.e., eight in total, the same number as in *Notothenia coriiceps* MTA (Figure 2b) or mammalian MT2s. (The lack of aromatic residues is a frequently cited feature of MTs, but we note that there are many exceptions to this rule: chicken MT and even some mammalian MTs do contain His residues, and in bacterial and some plant MTs, they participate crucially in metal binding [85].) The latter fact, together with the presence of only four carboxylate side-chains, is responsible for its relatively high theoretical isoelectric point of 8.23, very close to those of other vertebrate including fish MTs [34,36,53]. In (mammalian) MTs, lysines are known to stabilise the metal-thiolate clusters [86], which are overall negatively charged (−3 for M(II)_3_Cys_9_ and −1 for M(II)_4_Cys_11_). It may be worth noting here that precisely because of the presence of these charged metal-thiolate clusters, experimental isoelectric points differ considerably from the theoretical values, which do not take into account either metal ions or deprotonated cysteines.

Out of the 20 Cys residues in CgMT, 19 are in the same positions as those in non-fish vertebrate MTs. Only the 18th Cys is “shifted” from position 56 to position 54—a feature that is unique to fish MTs [36,87]. The solution structure of cadmium-loaded MTA from the icefish *Notothenia coriiceps* has been determined by ^1^H and ^113^Cd NMR spectroscopy [88]. Like other vertebrate MTs, the polypeptide chain folds into two largely independent globular domains, an N-terminal β-domain containing an M(II)_3_Cys_9_ cluster, and a C-terminal α-domain containing an M(II)_4_Cys_11_ cluster. The different location of a single Cys residue requires a change in the backbone conformation in the C-terminal region, and it has been suggested that this is a major determinant of the higher reactivity of fish MTs compared to mammalian MTs, when this is assessed under the exactly identical conditions. Another salient feature of fish MTs is their very high hydrophilicity [89]; this is also the case for CgMT, where its GRAVY (grand average of hydropathicity; the lower the GRAVY, the more hydrophilic the protein) value is −0.248. For comparison, this value is −0.005 for rat MT2. The higher hydrophilicity of fish MTs mainly results from an increase in serine and threonine residues at the cost of alanine and valine residues, along with a shift from an isoleucine to a valine residue (see Figure 2b). The higher hydrophilicity is also thought to increase conformational flexibility. Together, the higher flexibility and reactivity for fish MTs is thought to be a requirement arising from the fact that these animals operate at lower body temperatures than, e.g., mammals or reptiles [89].

Given the very high sequence similarity of CgMT with structurally characterised fish and mammalian MTs (Figure 2b), it can be suggested that CgMT adopts the same fold as these, and has similar metal-binding properties. In particular, one would expect that fully metal-loaded CgMT would have 7 M(II) (Zn or Cd) ions bound, and potential to bind up to 12 Cu(I) ions in two clusters [29,30,33,88]. To establish that the isolated cDNA sequence indeed codes for a functional protein, and to verify these predictions, we have recombinantly expressed CgMT in *E. coli*. The expression of eukaryotic MTs in this bacterial host has been carried out for scores of MTs, and, in many cases, it has been possible to synthesise and purify metallated protein [90,91,92,93]. This approach may be safer than in-vitro reconstitutions of apo-proteins, especially when no affinity tags are used for purification.

### 2.3. Expression and Metal-Binding Properties of Recombinant CgMT

MT proteins from fish have been quantified extensively, but studies involving biophysical characterisation are relatively rare [36,87,88,94,95].

In order to be able to synthesise CgMT protein in quantities sufficient for characterisation, *cgMT* cDNA was cloned into a pET-based expression vector, without any tags or surplus residues. DNA sequencing of the pET-*cgMT* expression construct confirmed that it contained the coding sequence of *cgMT* with no point mutations. The recombinant synthesis of CgMT was performed in the presence of zinc, cadmium, or copper. Whilst Zn(II)- and Cd(II)-bound CgMT were readily isolated from cell lysates in their metal-bound forms, we were repeatedly unable to recover sufficient protein from the copper-supplemented cultures using identical purification procedures.

**Figure 3:**
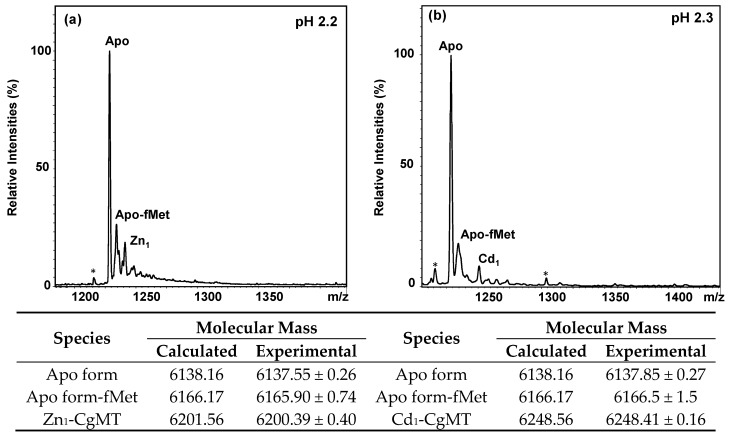
The most abundant +5 charge state from representative ESI-MS spectra of CgMT synthesised in the presence of: (**a**) Zn(II); or (**b**) Cd(II), recorded at acidic pH (10 mM NH_4_HCO_3_, 10% MeOH, 2% formic acid). Peaks marked with an asterisk (*) indicate impurities.

After purification of the metallated proteins by size-exclusion and anion-exchange chromatography, the identity and integrity of the CgMT protein were confirmed by Electrospray-Ionisation Mass Spectrometry (ESI-MS), after acidification of the purified protein solutions to pH 2.2–2.3. Across all preparations, a neutral molecular mass of 6137.70 ± 0.37 Da was found, in good agreement with the theoretical neutral mass of 6138.16 Da for the full 60-residue polypeptide (Figure 3). As should be the case [96], the N-terminal methionine was not cleaved; this residue is typically also present in vertebrate MTs isolated from the native host. In addition, each spectrum revealed the presence of two additional species: (i) The apo form of CgMT where the N-terminal methionine was still formylated (the average difference between the masses of the apo form and the fMet apo form, measured in individual spectra, was 27.11 Da, close to the expected 28.01 Da). This is a common feature of proteins overexpressed in *E. coli* [97]; (ii) There was a minor peak for a CgMT species with one metal ion bound (Figure 3). The fMet peak also overlaps with the peak for a mono-sodium adduct (+21.99 Da).

**Figure 4:**
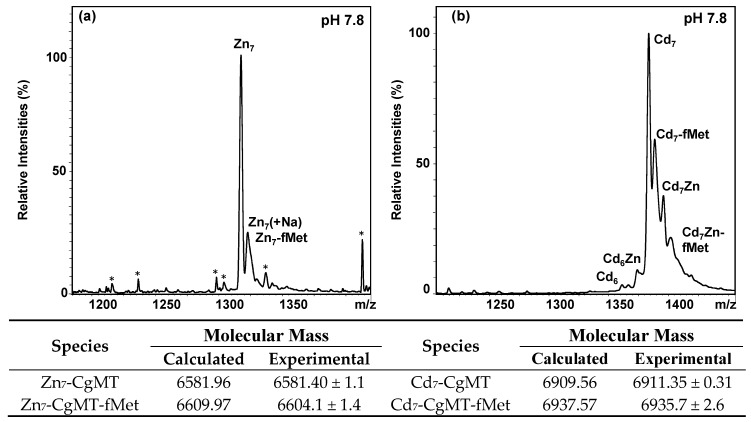
The most abundant +5 charge state of representative ESI-MS spectra of: (**a**) Zn(II)-CgMT; or (**b**) Cd(II)-CgMT (10 mM NH_4_HCO_3_, 10% MeOH) at native pH. The relatively large errors for the experimental masses for the fMet species are due to its low abundance and overlap with a sodium adduct that cannot be resolved for the metal-containing species due to their broad isotopic distributions. Peaks marked with an asterisk (*) indicate impurities.

Next, Inductively-Coupled Plasma Optical Emission Spectroscopy (ICP-OES) and ESI-MS were used to determine the metal-to-protein stoichiometry of metal-CgMT complexes. The ICP-OES results reported in Table 1 and the ESI-MS results reported in Figure 4 clearly demonstrate that for both Zn(II) and Cd(II) the expected [30,98] 7-M(II) species overwhelmingly dominate speciation at neutral pH. Interestingly though, preparations in presence of Cd(II) also contained over- (8 M(II)) and under- (6 M(II)) metallated species, and some Zn(II) was also bound to the protein.

**Figure 5:**
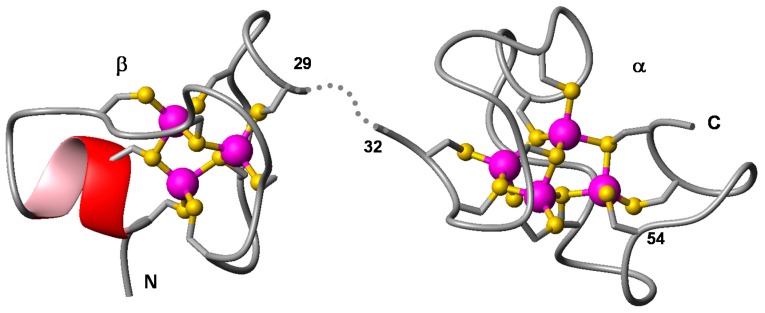
3D model for *C. gariepinus* MT showing the canonical M(II)_3_Cys_9_ (β domain) and M(II)_4_Cys_11_ (α domain) clusters. N- and C-terminus are indicated. The initial models were generated in PHYRE^2^ [102] and are based on pdb entries 1m0g and 1m0j (α and β domains of *Notothenia coriiceps* MTA, respectively [88]). The positions of the Cd(II) ions (magenta spheres) are also taken from these pdb entries. The two domains have been arranged in space by structural alignment with pdb entry 4mt2 (revised X-ray structure of rat liver MT [103]); the dotted line indicates the location for the linker residues 30 and 31, which are not resolved in the template NMR structures and hence also not in the model. The yellow spheres refer to thiolate sulphurs. The location of Cys54, which differs in all fish MTs from that of other vertebrate MTs, is also highlighted.

The fairly straightforward elemental analysis and mass spectrometry data, together with the high degree of conservation between CgMT and *Notothenia coriiceps* MTA (Figure 2) allowed generating a simple homology model for the Cd(II)_7_-CgMT species, depicted in Figure 5. The image illustrates the typical two-domain structure of vertebrate (and many other animal and plant) MTs. Apart from a short stretch of α-helix (residues Glu5-Thr9), no elements of regular secondary structure are present. The canonical M(II)_3_Cys_9_ and M(II)_4_Cys_11_ clusters determined for *Notothenia* MTA could be fitted effortlessly into the homology model.

In summary, the MT from *C. gariepinus* displays hallmarks of a typical Zn-MT [91]: with the divalent ions Zn(II) and Cd(II), it forms complexes with very well-defined stoichiometry, whilst in vivo in *E. coli*, copper could not stabilise the protein sufficiently to enable isolation of purified protein. In contrast, metal speciation studies on MTs isolated from, e.g., carp liver have indicated the existence of copper-bound MTs [99]. Notably, in the latter study, Cu and Zn co-eluted from the reverse-phase HPLC column, suggesting that mixed-metal complexes were present. It is thus possible that differences in metal metabolism between native and expression host are amongst the reasons for our inability to detect copper-bound MT. Furthermore, the capacity of rainbow trout MTA for binding Cu(I) has been demonstrated in vitro [94]. Although MTs can form more or less stable complexes with almost any heavy metal in vitro, Cu(I) forms, after Ag(I), the thermodynamically most stable complexes. Nevertheless, not all MTs yield defined Cu(I) complexes when synthesised in vivo, an observation that has given rise to the notion that MT sequences have evolved to “fit” their cognate metal ion(s) [100]. It is self-evident that trigonal planar or digonal Cu(I), the dominant oxidation state in the cytosol and the only form found bound to MTs, requires different backbone conformations than the tetrahedrally coordinated Cd(II) and Zn(II); it is also evident that different backbone folds have different stabilities—hence, it is conceivable that more stable folds, or less flexible proteins may be formed with different metals. Our difficulties in isolating significant quantities of MT from copper-supplemented cultures parallels those encountered previously with mammalian MT2 [101]. This may indicate that like the majority of vertebrate MTs (at least MT1 and MT2) [35], CgMT has evolved to primarily deal with Zn(II). Equally, like other vertebrate MTs, CgMT is also well-suited to bind Cd(II). Whether this may have implications for biological function is discussed further in Section 4.

With nucleotide sequence determined, and functionality of the protein established, we are now in a position to explore *cgMT*’s performance as a molecular biomarker.

### 2.4. Concentrations of Heavy Metals in Fish Liver Samples

**Figure 6:**
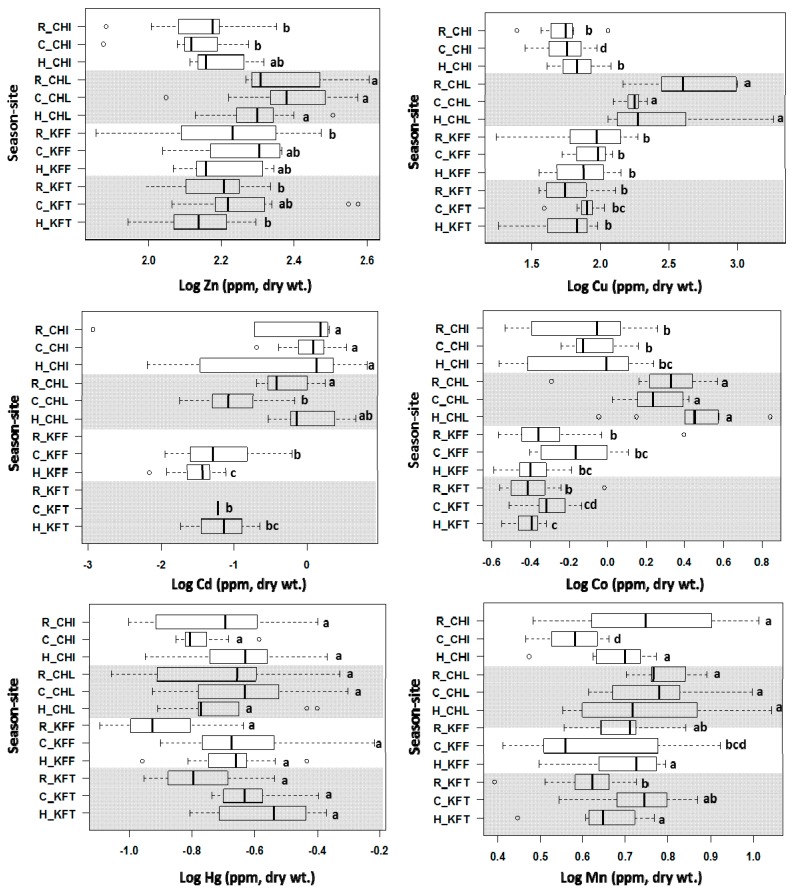
Levels of selected metals in liver tissue of *C. gariepinus* collected from four sample sites (CHI = Chimfunshi, CHL = Chililabombwe, KFF = Kafue Flats, KFT = Kafue Town) during three distinct seasons (R = warm-rainy season, C = dry-cold season, H = dry-hot season); sites are distinguished by different shading. One-way ANOVA followed by a post-hoc Tukey’s (HSD) test was applied to assess for spatial and seasonal differences in heavy metal levels. In the plots shown, levels for individual metals not connected by the same letter (“a”, “b” or “c”) are significantly different (*p* < 0.05) between sites, as analysed for each individual season. The ○ symbols in these box-and-whiskers plots denote outliers. Note that the logarithmic x axes scales are different for individual metals. In cases where no data are given, the respective metal was below detection limits. Also see Table S3 and Figure S4.

Our previous study on metal contents in sediments from the Kafue River [81] indicated that besides Cu, Co and Mn were also significantly enriched in sediments in the Copperbelt mining area, compared to the sediments in the unpolluted upstream reference site Chimfunshi (Table S1 and Figure S3 [81,104]). Zn and Pb were only slightly enriched, whereas Cr, Ni, Cd and Hg were less abundant in sediments from the mining area than in those from Chimfunshi. To establish whether the mining-related pollution is reflected in liver metal contents, we analysed the livers of 155 *C. gariepinus* fish caught in four different sites by Inductively-Coupled Plasma Mass Spectrometry (ICP-MS) after acid digestion of a small portion of each liver. Recoveries achieved by the chosen analytical method were determined using a certified reference material and ranged from 88% for Cu to 129% for Zn (Table S2). Analysis of heavy metal concentrations by sex did not show any significant differences and therefore data were not stratified by sex. Previous studies similarly concluded that sex had no influence on heavy metal accumulation in fish [105,106]. The full dataset is given in Table S3, and representative results are illustrated in Figure 6, with further plots compiled in Figure S4. The data are reported in ppm (μg·g^−1^), based on dry weight. The average water content of the livers was 74.12%.

Hg, Pb, Cr, Ni, and As levels in livers were low throughout, with As not detected at any of the study sites in any season. Ni was only detected at Chililabombwe in the warm-rainy season, and Pb was not detected in the warm-rainy season at any of the study sites. Overall, the mean heavy metal concentrations in liver samples at the study sites over the three seasons decreased in the order of: Fe > Zn > Cu > Se > Al > Mn > Co > Cd > Hg > Pb > Cr > Ni, As (Table S3).

The occasionally high variability of the results might be a consequence of variations in biotic factors such as age and size, which have been reported to have a moderate influence on heavy metal concentrations in fish [107]. In addition, up- or downstream migration of fish may result in contaminated fish caught in unpolluted sites, and vice versa [108]. Despite these complications, significant differences between sites are evident for several metals. In all three seasons, Cu and Co are significantly accumulated in livers of fish caught at Chililabombwe, the only site within the mining area where we were able to obtain fish. An increase in liver Zn levels is also evident at this site. The Co data are particularly clear, perhaps because normal Co concentrations in liver are much lower than either that of Zn or Cu, which are naturally already quite abundant in fish liver [109]. All three metals were previously detected at higher levels at this site in sediments compared to the other three sites [81] (Figure S3); therefore, it may be concluded that Cu, Co and perhaps also Zn are accumulated in livers as a consequence of pollution from mining activities. In contrast, corresponding differences in Mn levels in sediments were not reflected in Mn liver contents, which were fairly uniform around 5 ppm. It is likely that this different behaviour is due to differences in the homeostatic pathways for the different metals.

The comparatively high levels of Cd in sediments at Chimfunshi [81] were also reflected in increased Cd contents in livers in all three seasons. This is an interesting observation, because this site is considered pristine. It is possible that Cd concentrations in sediments, and consequently livers, are elevated due to the local geology and natural erosion of rocks, but further investigations are warranted. A trend towards higher Cd levels in fish livers is also apparent for Chililabombwe, compared to the Kafue Flats and Kafue Town sites, at least in the warm-rainy and dry-hot seasons. This could be a consequence of downstream migration of fish exposed to higher Cd levels at Chimfunshi and caught in Chililabombwe.

Figure 6 (and Figure S4) also allows observing seasonal variations of liver metal contents. Trends are hard to discern for either Chimfunshi or Chililabombwe, but further downstream at Kafue Flats and Kafue Town, the concentrations of Co, Cu, Zn, Cd, and Hg tended to be higher in the dry-cold season than in the rainy season. Such a trend has been observed previously [110]. Some heavy metals tend to accumulate more in fish samples in the dry-cold season due to the drought period which causes heavy metal salts to concentrate in water and ultimately accumulate in fish. Moreover, in the cold season most fish tend to settle at the bottom of the river for long periods of time and consume food from sediment which is the ultimate sink of heavy metals. In the case of Cd, Hg, and Pb, concentrations in the dry-hot season were also higher than in the warm-rainy season. Such increases in heavy metal concentration in fish samples in the dry-hot season can be related to an increase in river water temperature which increases the evaporation rate of surface water, hence, an increase of heavy metal concentrations in water and ultimately in fish [111]. Moreover, the metabolic rate of fish increases with temperature [112,113], resulting in more frequent feeding sessions which may result in increased heavy metal intake [114].

The cursory observations made above may be refined by conducting correlation analyses between the liver contents of the various metals across all sites and seasons (Table 2). Cu was most strongly correlated positively with Zn and Co, and these two metal ions are also strongly correlated with each other. Mn also correlated with Co and Cu, which, despite the very small variations in the liver Mn data, may yet be a consequence of the elevated Mn concentration in sediment at Chililabombwe. Pb was also correlated strongly with Cu and Co, but much more weakly with Zn and Mn. These five metals were all elevated in sediment samples at Chililabombwe (Figure S3), and we conclude that this may at least partially account for these correlations. Fe, which was also enriched in Chililabombwe sediments, correlated strongly with three of these metals, namely Co, Cu, and Zn, but not Mn or Pb. Cd correlated only strongly with Co; the origin of this correlation is unclear, although we note that both metals were at their lowest at Kafue Flats and Kafue Town. No strong correlations were observed for Hg, and the essential metalloid Se showed only weak correlations to Cu, Co, Zn, Cd and Pb.

Rationalising such correlation data for biological samples is not straightforward, as variations in metal contents in biological tissues do not only reflect sources of and pollution from individual metals, but also differences in metal metabolism. To investigate to what extent sediment was a major source for individual metals accumulated in *C. gariepinus* livers, a correlation analysis between metal contents in fish liver samples and river sediments collected at the same sites and at the same times (see Section 2.1; data taken from [81]) was carried out (Table S4). Correlations were observed for Al, Mn, Cu, Co, and Cd, indicating that, where these metals were elevated in fish livers, sediment was likely an important source of these metals, presumably due to the bottom-feeding habits of *C. gariepinus*. The correlations for Cu and Co were strongest; this is mainly a consequence of the clearly elevated levels in sediments and livers at Chililabombwe. The correlation for Zn is much weaker; this may be a consequence of this metal being only slightly enriched in sediments at Chililabombwe, normal liver levels of Zn being relatively high in any case, and the homeostasis of the essential Zn being very well controlled. Interestingly, the correlation values for toxic Pb were similar to those for Zn. Pb was also slightly enriched in sediments at Chililabombwe (Figure S3), explaining the existence of a correlation. No significant correlations were observed for Cr, Fe, Se, and Hg; thus, the composition of the sediments was not a major factor in determining the levels of these elements in fish livers.

In summary, the metal analysis data indicate clearly that fish at Chililabombwe have elevated levels of Cu, Co, Fe, Zn and Pb (Figure 6 and Figure S4) in their livers, and that for Co and Cu, this is at least in part a consequence of higher enrichment in sediments (Tables S1 and S5, and Figure S3). We next explored whether variations in liver metal contents are reflected in MT expression to test the utility of *cgMT* expression as a biomarker for metal pollution.

### 2.5. MT Gene Expression Levels in Liver of C. gariepinus

**Figure 7:**
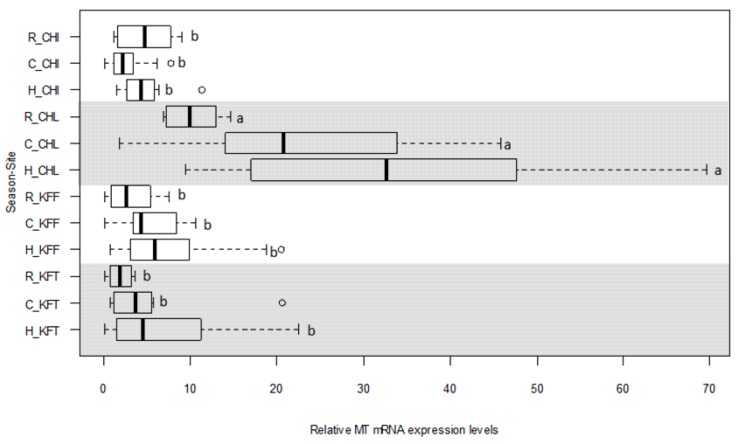
*CgMT* mRNA expression levels in liver tissue of *C. gariepinus* collected from 4 sample sites (CHI = Chimfunshi, CHL = Chililabombwe, KFF = Kafue Flats, KFT = Kafue Town) during three distinct seasons (R = warm-rainy season, C = dry-cold season, H = dry-hot season). *CgMT* levels were determined by qPCR (7500 Fast Real-Time PCR System, Applied BioSystems) from each season/site condition, each of which consisted of 10-18 individual biological replicates (see Table S3). *CgMT* quantification was performed using three technical replicates per sample and was normalised to *β-actin*. One-way ANOVA followed by a post-hoc Tukey’s (HSD) test was applied to assess for spatial and seasonal differences in MT expression levels. Different letters (“a” and “b”) indicate a statistically significant difference (*p* < 0.05) between sites in each season. The ○ symbols in this box-and-whiskers plot denote outliers.

The same 155 livers as analysed for heavy metal contents were used to quantify the relative expression of *cgMT* in the liver of *C. gariepinus* (Figure 7). Although some studies have reported sex-dependent differences in MT expression levels [28], no sex difference was apparent in *cgMT* expression levels in the livers used in our study (data not shown), and therefore data were not stratified by sex.

*CgMT* expression levels, expressed relative to the sample that showed the lowest level (collected from Chimfunshi), ranged from 2.00 (Kafue Town in the warm-rainy season) to 34.2 (Chililabombwe in the dry-hot season). *CgMT* expression levels decreased by site and season in the order: Chililabombwe (dry-hot season) > Chililabombwe (dry-cold season) > Chililabombwe (warm-rainy season) > Kafue Flats (dry-hot season) > Chimfunshi (warm-rainy season) > Kafue Town (dry-hot season) > Chimfunshi (dry-hot season) > Kafue Flats (dry-cold season) > Kafue Flats (warm-rainy season) > Chimfunshi (dry-cold season) > Kafue Town (warm-rainy season). Hence, the Chililabombwe site in the mining area showed the highest *cgMT* transcript levels in all three seasons. No significant site differences in *cgMT* expression levels were observed amongst fish from Chimfunshi (site upstream from the Copperbelt mining area), Kafue Flats and Kafue Town (sites far downstream). Compared to Chimfunshi, *cgMT* mRNA expression levels in fish from Chililabombwe were increased approximately 2.2-fold in the warm-rainy season, 8.9-fold in the dry-cold season and 7.1-fold in the dry-hot season. Considering seasonal variations, there is a trend towards lower expression levels in the warm-rainy season (except at Chimfunshi), similar to the trends observed for most metal ions (Section 2.4, Figure 6).

Hence, given the elevated levels of metals in the livers from Chililabombwe, and similarities in seasonal trends (Figure 6 and Figure S4, and Table S3), it can be qualitatively confirmed that in the catfish *C. gariepinus*, elevated metals in liver are indeed reflected in hepatic MT expression. A more quantitative picture may be obtained by conducting a correlation analysis between *cgMT* expression levels and heavy metals in liver tissue across all seasons, carried out separately for each of the four sites (Table 3). A correlation analysis across all sites and seasons can be found in Table S5.

As has been pointed out [20], in field studies, the bioindicator organism is typically exposed to mixtures of metals, or indeed other pollutants, making it impossible to attribute MT induction to (a) particular metal(s). For the following considerations, it is therefore imperative to bear in mind that the data in Table 3 do not provide an analysis of whether or not *cgMT* responds to the levels of a particular metal ion, but at most which metal ion(s) govern the expression of *cgMT* under the prevailing field study conditions. The most fundamental hypothesis that may underlie the data in Table 3 states that the presence of a particular metal induces MT transcription. It has been known for a long time that many metals can induce MT transcription, including Zn, Cu, Cd, Hg, Pb, Mn, Fe, Ni, Co [28,30,40,50,115,116] and As [117]. In contrast, selenite may downregulate MT expression [118]. Thus, in principle, almost any of the metal ions under study might contribute to MT expression levels.

Clearly, in the non-polluted site Chimfunshi, the only significant correlations observed were those with liver Zn and Cu. Strong correlation with Zn was also observed at Kafue Town and Kafue Flats, but this was completely lost in Chililabombwe, the site within the mining area, even though the levels of Zn in livers were elevated in fish from this site (Figure 6). It remains an interesting question whether these elevated levels of Zn in the livers from Chililabombwe are cause or consequence of elevated MT protein levels, as MTs are known to contribute to metal retention [119].

These data display various parallels with literature reports, for example, positive correlations with hepatic Cu levels were reported for perch [120], rainbow trout [48], eel [55,107], golden-grey mullet [121], and gilt-head bream [60]. Correlations with hepatic Zn also have been observed frequently, for example also in eel [55], striped red mullet [121] and feral perch, gudgeon and roach [122]. Given that Zn and Cu also are two of the metals most frequently found associated with MTs in vivo [30,99], these correlations are within expectations, irrespective of our inability to detect copper-binding forms of CgMT in our recombinant system.

In addition, correlations were observed for Cd and Se at Chililabombwe, and for Hg at Kafue Flats and Kafue Town. Cd has been repeatedly proven to be a strong inducer of MT expression, including in fish [24,36,55,60,115]. The Chililabombwe site did not have particularly high average Cd levels, neither in sediments, nor in livers (Tables S1 and S3). Since the analysis in Table 3 correlates data for individual fish, it is possible that the liver Cd levels differed significantly between fish depending on their life history, and that elevated Cd was an additional contributor to increased liver *cgMT* expression. The origin of the strong positive correlation with Se at Chililabombwe is unclear. It is unlikely to be related to pollution with selenium, but elevated liver selenium might be a consequence of copper-induced oxidative stress leading to upregulation of antioxidant selenoproteins [123].

Although average Hg values at Kafue Flats and Kafue Town were not significantly elevated in fish livers compared to those from the other sites (Figure 6 and Table S3), correlations between liver Hg levels and MT expression were apparent within these two sites. The plot for Hg in Figure 6 hints at seasonal differences in liver Hg levels at Kafue Flats and Kafue Town, with a trend towards lower levels in the warm-rainy season. Mercury exposure leading to MT induction has been observed previously [124,125,126]. Whilst the average values did not display statistically significant differences, it appears possible that small seasonal variations in Hg levels may be contributing to MT induction levels, and this trend should be followed up in the future.

No correlations were observed for Al, Cr, Mn, Fe, Co, Ni and Pb. With the exception of Al, all of these metals have been reported to induce MT synthesis in various organisms [40,115,116,127,128,129]. The absence of correlations with these metals in our study has various causes: the levels of Al, Cr, Mn, Ni and Pb are low and fairly invariant; those of Fe are high throughout; and, although Co is elevated at Chililabombwe (Figure 6), the per-site correlation analysis shows that, within a given site, this metal is not a major contributor to MT induction in the given background of high Cu. If data are analysed across all sites (Table S5), a correlation with Co is apparent (along with weaker correlations for all metals that are elevated in livers from this site); this is due to elevated liver Co levels at Chililabombwe, which coincide with elevated *cgMT* expression.

In summary, expression of MT in wild *C. gariepinus* correlates with liver Cu and Zn levels in non-contaminated conditions, whilst extraordinarily high levels of Cu abrogate the correlation with Zn. Further correlations with low levels of toxic Cd and Hg indicate that more subtle pollution may also be picked up, warranting future studies.

### 2.6. Suitability of C. gariepinus Liver MT mRNA as a Biomarker

To ultimately assess whether *cgMT* expression can be used as a biomarker of pollution, we have carried out further Pearson correlation analyses to explore links between metals in sediment and *cgMT* expression (Table S6). Inclusion of all four sites in the analysis shows that *cgMT* expression correlates with all metals that are elevated (Mn, Co, Cu, Zn, and Pb; Figure S3) in sediments at the polluted site Chililabombwe.

Pollution and ecological risks are always consequences of mixtures of pollutants; therefore, quantitative indices that summarise overall pollution and its impacts are defined in Ecotoxicology [130]. A successful biomarker may be expected to also correlate well with such cumulative indices. To explore this expectation, we have calculated correlations between *cgMT* expression, the “pollution load index” (PLI), and the “potential exological risk index” (RI). PLI and RI values are reported in reference [81], and were derived from the sediment metal data for each site/season combination apart from the uncontaminated site Chimfunshi, which served as reference site. (PLI values are generally calculated from the contamination factors for each metal (CF = concentration(site)/concentration(reference site)), using the following relationship [131]: PLI = CF(metal1) × CF(metal2) × …)^1/n^. RI values are calculated from the sum of the potential ecological risk factors (Er = Tr × concentration(site)/concentration(reference site)) for each metal, where Tr is the “toxic response factor” and depends on each metal’s toxicity (for example, Tr(Hg) = 40, and Tr(Zn) = 1 [132]).) PLI aims to give a summary reflection of pollution from a mixture of contaminating metals, whilst RI concerns their impact and thus takes into account their toxicity. PLI and RI values for each of the nine site/season combinations are tabulated in Table S7 (compiled from reference [81]). Liver MT expression in fish caught at these sites in each of three seasons was strongly correlated with pollution as expressed by the PLI (*r* = 0.45; *p* < 0.0001) as well as with potential ecological risk (*r* = 0.67; *p* < 0.0001 for RI). Both correlations are largely a reflection of the high levels of pollution from Cu at Chililabombwe (see also Table S4).

In summary, despite considerable variations in the data for both metal levels and MT expression, probably due to a range of biotic factors, the elevation of *cgMT* expression in fish from the polluted Chililabombwe site was unequivocal. We conclude that, within the context of our cross-sectional study, *cgMT* expression in the livers of *C. gariepinus* fish, as measured by quantifying mRNA, can serve as a suitable biomarker of pollution and ecological risk, and could be developed into a sensitive tool for biomonitoring purposes, especially in the context of mitigating the environmental effects of mining activities in the Copperbelt region.

## 3. Materials and Methods

### 3.1. Fish Collection, Preparation and Sampling

*C. gariepinus* weighing between 600 to 1200 g were sampled in the warm-rainy, dry-cold season and dry-hot seasons in 2014. A minimum of 10 individual fish per site in each season were randomly purchased from fishermen and initially stratified according to sex. Each fish was humanely killed and thoroughly cleaned with distilled water before dissection. A ventral midline incision was made and liver samples were dissected. Each of 155 livers was used in heavy metal determination and MT expression analysis. Samples for heavy metal analysis were placed in aluminium foil. For the determination of metallothionein mRNA levels, about 1 cm^3^ of each liver was placed in an Eppendorf tube containing 1 mL RNAlater^®^ (Sigma Aldrich, Irvine, UK). All samples were frozen and transported in liquid nitrogen and then stored at −80 °C until analysis.

### 3.2. Total RNA Isolation and Reverse Transcription Reaction

Total RNA was isolated from liver using TRI Reagent (Sigma Aldrich) according to the manufacturer’s protocol. About 18 to 20 mg of liver tissue was crushed in 1 mL of TRI Reagent using a hand-held RNase-free pellet pestle (VWR International Limited, Lutterworth, UK) until the tissue was homogenised. The quantity and integrity of isolated RNA was checked on 1% agarose gel stained with GelRed™ Nucleic Acid Gel Stain (VWR International Limited) in 1× Tris-acetate-EDTA (TAE) buffer and by spectrophotometric measurement using NanoDrop Lite (ThermoFisher Scientific, Rugby, UK). Total RNA was stored at −80 °C until use.

The cDNA was synthesised from total RNA using the RevertAid First Strand cDNA Synthesis Kit (ThermoFisher Scientific). The 12 µL reaction mixture contained: 3 µg of total RNA, 1 µL of 100 µM oligo(dT)_18_ primer and nuclease free water (ThermoFisher Scientific). The mixture was incubated at 65 °C for 5 min, and then at 0 °C for 2 min. Thereafter, 4 µL of 5× RT Buffer, 20 units of RiboLock RNase inhibitor, 2 µL 10 mM dNTPs and 200 U of RevertAid^TM^ Premium Reverse Transcriptase were added. The reaction mixture was incubated at 42 °C for 60 min and terminated at 70 °C for 10 min. The obtained cDNA was stored at −20 °C until analysis.

### 3.3. Primer Design and Reverse Transcription Polymerase Chain Reaction (RT-PCR)

In order to amplify cDNA encoding *C. gariepinus* metallothionein (*cgMT*), degenerate primers were designed using sequences deposited in GenBank at NCBI encoding MTs from closely related species in the same order as *C. gariepinus*—Siluriformes: *Clarias macrocephalus* (accession No JX312865.1) and *Pelteobagrus fulvidraco* (accession No FJ418583.1). The primers were as follows: forward primer 5′-ARYKWSMTYTWTTTGRAAAGCGA-3′ and reverse primer 5′-YMWSRTGMYRRKARTSTTGGAGT-3′ (IDT Integrated DNA Technologies, Leuven, Belgium), where R = A or G, Y = C or T, K = G or T, W = A or T, S = G or C and M = A or C. The product size was around 200 bp. In addition, to check the *C. gariepinus* β-actin (*cgβ-actin*) sequence, a partial cDNA for this gene was also generated, using primers designed based on its sequence available at GenBank (accession No KJ722167.1). The expected product with a size of 396 bp was amplified with forward 5′-GATGATGAAATCGCCGCACT-3′ and reverse 5′-ATACATGGCTGGGGTGTTGA-3′ primers (IDT Integrated DNA Technologies). To amplify *cgMT* and *cgβ-actin* cDNA, the RT-PCR reactions were prepared using the Phusion High-Fidelity PCR Kit (ThermoFisher Scientific). The reaction mixture included: 1.5 µL of single-stranded cDNA as template, 0.4 µL of 10 µM forward and 0.4 µL of 10 µM reverse primers, 0.4 µL of 10 mM dNTPs, 4 µL 5× buffer, 0.4 U of Phusion High-Fidelity DNA Polymerase and nuclease free water (ThermoFisher Scientific) in a total volume of 20 µL. The cycling conditions were as follows: initial denaturation: 98 °C for 30 s; denaturation: 98 °C for 10 s, annealing: 52 °C for 40 s and extension: 72 °C for 40 s for 30 cycles; final extension at 72 °C for 5 min. The PCR products were visualised on a 2% agarose gel. PCR products were extracted from the gels using QIAquick Gel Extraction Kit (Qiagen, Hilden, Germany) according to the manufacturer’s protocol. The purified PCR products were then ligated with pJET1.2 blunt vector (CloneJET PCR Cloning Kit, ThermoFisher Scientific) according to the manufacturer’s protocol and sent to GATC Biotech (Konstanz, Germany) for sequencing.

### 3.4. Expression and Purification of Recombinant CgMT

PCR was performed to amplify the open reading frame of *C. gariepinus* metallothionein on the pJET1.2 plasmid bearing the partial cDNA of *cgMT* using sequence-specific primers (IDT Integrated DNA Technologies, Leuven, Belgium) containing an *Nde*I (forward) or an *Xho*I (reverse) restriction site, respectively: forward 5′-AAACATATGGACCCCTGCGAGTGTTCAAAG-3′ and reverse 5′-AAACTCGAGTCACTGACAGCACTTGGAATCAC-3′ (restriction sites are underlined). The PCR mixture contained: 1.5 µL plasmid DNA as a template, 0.6 µL of 10 µM forward and 0.6 µL of 10 µM reverse primers, 0.4 µL of 10 mM dNTPs, 4 µL 5× buffer, 0.4 U of Phusion High-Fidelity DNA Polymerase (ThermoFisher Scientific) and nuclease free water (ThermoFisher Scientific) in a total volume of 20 µL. The thermal cycling conditions were as follows: initial denaturation: 98 °C for 30 s; denaturation: 98 °C for 10 s, annealing: 52 °C for 30 s and extension: 72 °C for 30 s for 30 cycles; final extension at 72 °C for 5 min. After digestion with *Nde*I and *Xho*I, the PCR product was ligated into pET21a(+) bacterial expression vector (Novagen, Darmstadt, Germany). In order to confirm that the open reading frame was inserted correctly and that there were no mutations, sequencing of the isolated plasmids was performed (GATC Biotech). The obtained construct was named pET-CgMT.

CgMT was recombinantly expressed in *E. coli* Rosetta 2 (DE3) pLysS (Novagen) in the presence of Zn^2+^, Cd^2+^ or Cu^2+^ (the latter under low aeration to promote levels of Cu(I)) [91]. Overnight cultures of transformed bacteria were diluted 1:100 (*v*/*v*) in LB medium containing chloramphenicol (34 µg/mL) and ampicillin (100 µg/mL). When the optical density of the culture had reached ca. 0.6–0.7 at 600 nm, the expression of protein was induced by adding 0.5 mM isopropyl β-d-1-thiogalactopyranoside (IPTG; Sigma-Aldrich, Irvine, UK) and ZnSO_4_ to a final concentration of 0.5 mM, CdSO_4_ to a final concentration of 0.3 mM, or CuSO_4_ to a final concentration of 0.1 mM. The cultures were incubated at 15 °C overnight before harvesting by centrifugation. The cell pellet was suspended in lysis buffer (50 mM Tris, 100 mM KCl, 3 mM DTT, 0.5% Triton X-100 and either Zn^2+^ or Cd^2+^ to a final concentration of 1 mM as appropriate to the metal added during protein expression, pH 8.5) and cells were disrupted using a cell disruptor (Constant Systems Limited, Daventry, UK) at a pressure of 20 kpsi. Cell lysate was separated from cell debris by centrifugation at 11,000 rpm and 4 °C for 30 min, filtered through Millipore syringe filters (0.22 µM) and purified by fast protein liquid chromatography (FPLC) (GE Healthcare Äkta Purifier, Little Chalfont, UK) using a size exclusion column (SEC) (HiLoad 16/60 Superdex 75, Amersham Biosciences, Little Chalfont, UK) equilibrated with 20 mM NH_4_HCO_3_ buffer. For further purification, fractions from SEC were combined and loaded onto an anion exchange column (AEX) (HiTrap Q XL 5 mL, GE Healthcare, Little Chalfont, UK). The column was washed with 25 mL of high salt buffer (1 M NaCl, 20 mM NH_4_HCO_3_, pH 9.0) followed by equilibration with 25 mL of low salt buffer (20 mM NH_4_HCO_3_, pH 9.0). The elution of the protein was monitored by measuring absorbance at 220 and 280 nm. Selected fractions were pooled and desalted using PD10 desalting columns (Sephadex G-25, GE Healthcare) with 10 mM NH_4_HCO_3_ buffer. Next, eluates from PD10 columns were analysed for metal and sulphur contents by inductively-coupled plasma optical emission spectroscopy (ICP-OES, Perkin-Elmer Optima 5300 DV, Model S10, Llantrisant, UK; vide infra), and/or analysed on SDS-PAGE gels. Proteins were resolved on precast 4–15% Mini-PROTEAN^®^TGX™ Gel (Bio-Rad, Watford, UK) in TGS (Tris/Glycine/SDS, pH 8.3; Bio-Rad) buffer and visualised by silver staining. Protein concentration was determined via measuring S content by ICP-OES or by measuring thiol content using Ellman’s reaction [133] after demetallation with 1 mM EDTA.

### 3.5. Analysis of Metal-Binding Stoichiometries of Recombinant CgMT

The contents of S, Zn and Cd in samples were determined by ICP-OES by measuring S at 181.980 and 180.675 nm, Cd at 228.806 and 214.449 nm and Zn at 206.204 and 213.863 nm. Mixed element calibration standards were prepared in the range of 0.2–5 ppm from commercial stocks (TraceCERT, Sigma-Aldrich). All samples and standards were prepared in 0.1 M HNO_3_ (ultrapure 70% HNO_3_ purified in-house by sub-boiling point distillation of reagent-grade HNO_3_).

Prior to analysis by electrospray ionisation (ESI) time-of-flight mass spectrometry (MicrOTOF, Bruker Daltonics, Bremen, Germany), samples were concentrated to 20–30 µM using Amicon Ultra-4 3000 Da MWCO centrifugal filters. The parameters for ESI-MS were as follows: source temperature 468 K, mass spectral voltage parameters: 210 V capillary exit, 450 V hexapole RF, 1:70 V skimmer, 1:19 V hexapole. MS data were acquired in the positive mode in the range of 500–3500 mass to charge (*m*/*z*) ratio with an average mass spectrum being generated from 2 min of analysis time. For the mass spectra of metallated proteins, the samples in 10 mM NH_4_HCO_3_ at pH 7.8, were mixed with 10% (*v*/*v*) methanol and injected directly into the spectrometer using a syringe pump at a flow rate 240 µL/h. In order to obtain mass spectra for the apo form of the protein, concentrated formic acid was added to give a pH of ca. 2. Deconvoluted spectra were generated from the most intense peak in the raw spectra and were analysed using Data Analysis 4.0 software (Bruker Daltonics, Bremen, Germany).

### 3.6. Quantitative Real-Time PCR

MT mRNA levels were determined by quantitative real-time PCR (7500 Fast Real-Time PCR System, Applied BioSystems, Foster City, CA, USA) using ROX qPCR master mix (ThermoFisher Scientific). Custom primers and probes (Sigma Aldrich) for *cgMT* and *cgβ-actin* were designed using ProbeFinder Assay design Software of the Universal ProbeLibrary (Roche Life Science, Indianapolis, IN, USA) (Table 4).

The reaction mixture contained the following: 2 µL cDNA (1:10), 0.8 µL of 10 µM forward primer, 0.8 µL of 10 µM reverse primer, 0.2 µL probe, 10 µL of 2× ROX qPCR master mix (ThermoFisher Scientific) and nuclease free water (ThermoFisher Scientific), to make 20 µL. Thermal cycler conditions were as follows: incubation stage at 50 °C for 2 min, initial denaturation at 95 °C for 10 min, denaturation at 95 °C for 15 s, annealing and extension at 60 °C for 1 min for 40 cycles. Negative controls were included in all the runs to confirm the absence of contamination. Additionally, to confirm that the amplification was only of synthesised cDNA and not of contaminating genomic DNA, after amplification, samples were analysed by electrophoresis on a 1% agarose gel. Since the two primers were designed to anneal to two different exons (Figure S5), amplification of genomic DNA would have led to larger PCR products, but no such larger PCR products were observed. All determinations are the result of three replicates.

Relative quantification of MT mRNA levels was carried out by standardising gene expression against the house-keeping gene *cgβ-actin*, using the comparative ∆∆*C*_t_-method. A sample from the Chimfunshi site that showed the lowest *cgMT* expression levels was chosen as the control sample, and calculation of expression levels of *cgMT* in each of the remaining 154 samples followed Equation (1):

*cgMT* fold change = 2^−((MT exposure − β-actin exposure) − (MT control − β-actin control))^ (1)

### 3.7. Heavy Metal Analysis in Fish Liver

All laboratory equipment required for heavy metal analysis were pre-soaked in 2% (*v*/*v*) HCl (ThermoFisher Scientific) overnight and rinsed twice with ultrapure water (18 MΩ·cm; Millipore Milli-Q, Watford, UK). All chemicals were analytical grade reagents, and Milli-Q water was used for the preparation of all solutions.

The liver samples were freeze-dried (VirTis, BenchTop K, SP Scientific, Gardiner, NY, USA) for 24 h and were acid digested following the Milestone DG-FO-17 digestion method for dried fish with some modifications. Approximately 0.5 g of each freeze dried liver sample was weighed, ground and homogenised and then transferred into a Teflon digestion vessel (Milestone Inc., Shelton, CT, USA). Metals were extracted using a closed microwave digestion system (Start D, Milestone) after addition of 7 mL of ultrapure 72% (*v*/*v*) HNO_3_ (prepared in-house from reagent grade HNO_3_ by sub-boiling-point distillation) and 1 mL of 30% (*v*/*v*) H_2_O_2_ (Primar-Trace analysis grade, ThermoFisher Scientific) to the digestion vessels. The digestion programme and temperature profile was as follows: step 1 (ramp time)—1200 W at 180 °C for 10 min, step 2 (hold time)—1200 W at 180 °C for 30 min.

The digested samples were then diluted 31.25 fold with ultrapure water, keeping the contact time with glass to a minimum. The samples were analysed for total metal concentrations using the ^27^Al, ^53^Cr, ^55^Mn, ^56^Fe, ^59^Co, ^60^Ni, ^63^Cu, ^66^Zn, ^75^As, ^82^Se, ^111^Cd, ^202^Hg and ^208^Pb isotopes and a 7500 series ICP-MS (Agilent Technologies, Stockport, UK) equipped with a cross flow nebuliser, a quartz spray chamber, and an Octopole Reaction System (ORS^®^) cell. The instrument was operated in helium gas-mode to remove matrix interferences. A multi-element standard solution IV for ICP and a single mercury standard for ICP (Fluka Analytical, Buchs, Switzerland) were used to prepare seven multi-element standards (^27^Al, ^75^As, ^208^Pb—0.44 to 800 ppb, ^53^Cr, ^60^Ni, ^63^Cu—0.20 to 400 ppb, ^56^Fe, ^66^Zn, ^82^Se—1.00 to 2, 000 ppb, ^55^Mn, ^59^Co, ^111^Cd—0.10 to 200 ppb and ^202^Hg—0.02 to 35.0 ppb) to provide suitable calibration curves. Only calibration curves with *r*^2^ > 0.999 were accepted for concentration calculations. Erbium (^166^Er) was used as an internal standard. For quality control, sample replicates were used to assess precision of the analyses, and reagent blanks and certified reference materials (ERM^®^—BB422, fish muscle European Commission; Sigma-Aldrich, Irvine, UK) were used to assess method accuracy. Hence, each ICP-MS run included triplicate blanks, standards, triplicate CRM and triplicate samples. The detection limits for heavy metal analysis (ppb) were: Al (1.203), Cr (0.05853), Mn (0.02010), Fe (0.7638), Co (0.01884), Ni (0.06114), Cu (0.1284), Zn (0.2880), As (0.01216), Se (1.208), Cd (0.03024), Hg (0.008549) and Pb (0.006629).

### 3.8. Statistical Analyses

Descriptive statistics were calculated using Microsoft Excel^®^ 2013. Further statistical analyses were performed using R (R Core Team, 2014, Vienna, Austria) after the data were normalised by log_10_ transformation. The mean values of concentrations of heavy metals and CgMT mRNA expression levels were then subjected to one-way analysis of variance (ANOVA) to test for spatial and seasonal differences (*p <* 0.05). Where significant differences were present, the mean values were separated using post-hoc Tukey’s (honest significant difference) test. Pearson correlation analysis was performed to test for inter-metallic associations and associations between heavy metals and *cgMT* gene expression levels (*p <* 0.05). Welch two sample *t*-test was used to test for sex differences in heavy metal accumulation and *cgMT* gene expression levels (*p <* 0.05).

## 4. Conclusions

This study established that *C. gariepinus* from the Kafue River experienced heavy metal contamination that varied depending on site and season. The potential of MT as a biomarker of heavy metal pollution in *C. gariepinus* from the Kafue River was demonstrated, especially for Cu. This information will be useful for environmental agencies and legislators to make rational decisions regarding further use of *cgMT* as biomarker, heavy metal pollution of fish from the Kafue River, and the usage of these fish for human consumption.

In vitro biophysical analysis of the recombinantly expressed CgMT protein revealed typical “Zn-thionein” [91] character, very similar to mammalian MT2. The strong correlation of *cgMT* expression with Cu observed in the field study, and our inability to isolate Cu-bound CgMT are not as contradictory as it may seem, but give rise to the opportunity to consider the following observations and comments.

(i) In the ecotoxicological literature concerning MTs, there seems to be a widespread assumption that increased levels of MT protein provide protection against (acute) metal toxicity by sequestering the respective metal. Broadly speaking, metal toxicity can be mediated by adventitious binding of excess or toxic metal ions to biomolecules and/or by oxidative stress caused by redox active metal ions, primarily by generating reactive oxygen species, or by blocking reduced thiols. Accordingly, owing to their coordination and redox chemistry, MTs are in principle able to counteract both these modes of acute metal toxicity. It must be stressed though that there is no reason to claim that induction by a particular metal ion equates to in vivo binding or sequestration, unless this has been demonstrated experimentally, e.g., by appropriate metalloproteomics experiments on the native organism. Induction or even complex formation with a particular metal in vitro is insufficient to support such claims. Furthermore, Bourdineaud et al*.* have pointed out that MT induction may primarily serve to compensate for lysosomal degradation of existing MT protein, rather than necessarily leading to increased protein levels [49]. Together with the observation that toxic metal levels in cells can be easily higher than the total MT binding capacity, this argues against a simple sequestration role. Nevertheless, MTs *can* be one part of the machinery to deal with elevated or toxic metals. The fact that Cu-MT association has been demonstrated very convincingly in fish liver cytosols even in control fish [99] suggests that at least some of the Cu enriched in *C. gariepinu*s livers was bound to MT.

(ii) There is also the widespread assumption that heavy metal detoxification is part of the repertoire of biological functions of MTs. We note that there is some controversy regarding this, especially as far as vertebrates are concerned [35]. Even where toxic metal-MT interactions in vivo have been confirmed, an evolutionarily determined biological function cannot necessarily be inferred. Instead, it has been argued that in particular MT induction in response to cadmium exposure and in-vivo cadmium-binding are “properties” rather than functions of mammalian MTs [35]. Nevertheless, in organisms that are naturally exposed to these metals in soil or water, metal detoxification may well be an evolved function for MTs. The case for cadmium handling is particularly clear in some terrestrial invertebrates [93,134,135]. The situation in fish is unclear, but, given that fish inhabit environments where cadmium levels under normal circumstances are only two to three orders of magnitude lower than those of essential zinc, it is in principle possible that dealing with cadmium could be a true function of fish MTs, even though this was not apparent in our field study.

## Figures and Tables

**Table 1:** Metal stoichiometry of recombinant CgMT protein expressed in presence of Zn(II) or Cd(II), as determined by elemental analysis (ICP-OES).

Supplemented Ion	Sulphur (mg/L)	Sulphur (µM)	Protein (µM)	Metal	Metal (mg/L)	Metal (µM)	M: Protein Ratio
Zn(II)	0.78	97.81	4.66	Zn	0.54	33.06	6.88 ± 0.89
Cd(II)	2.31	144.38	6.88	Cd	2.98	53.01	7.72 ± 0.83

**Table 2:** Inter-metallic correlation coefficients (lower left-hand side) and *p*-values (in parentheses, upper right hand side) in fish liver samples at all four study sites over the three seasons. Significant correlations are highlighted with an asterisk (* *p* < 0.05) and in bold (*p* < 0.00001).

	Al	Cr	Mn	Fe	Co	Cu	Zn	Se	Cd	Hg	Pb
Al	-	(0.5719)	(0.0426 *)	(0.1794)	(0.6672)	(0.7432)	(0.2225)	(0.2097)	(0.0087 *)	(0.1707)	(0.3160)
Cr	−0.15	-	(0.7745)	(0.5747)	(0.7506)	(0.4018)	(0.7898)	(0.5735)	(0.0380 *)	(0.8042	(0.6222)
Mn	0.18	−0.08	-	(0.0169 *)	(<0.00001 *)	(<0.00001 *)	(0.0032 *)	(0.2207)	(0.0285 *)	(0.0056 *)	(0.0010 *)
Fe	0.12	−0.15	0.22	-	(<0.00001 *)	(<0.00001 *)	(<0.00001 *)	(0.1127)	(0.8114)	(0.0883)	(0.2067)
Co	−0.04	−0.08	**0.45**	**0.38**	-	(<0.00001 *)	(<0.00001 *)	(0.0005 *)	(<0.00001 *)	(0.0058 *)	(<0.00001 *)
Cu	0.03	0.22	**0.36**	**0.41**	**0.66**	-	(<0.00001 *)	(0.0019 *)	0.0290 *)	(0.0344 *)	(<0.00001 *)
Zn	0.11	0.07	0.26	**0.60**	**0.41**	**0.66**	-	(0.0453 *)	(0.4079)	(0.0017 *)	(0.1168)
Se	−0.11	0.15	0.11	0.14	0.31	0.28	0.18	-	0.0003 *)	(0.8902)	(0.0319 *)
Cd	−0.29	0.54	0.24	0.03	**0.56**	0.24	−0.09	0.39	-	(0.6306)	(0.0109 *)
Hg	0.13	(0.07)	0.25	0.15	0.25	0.19	0.28	0.01	0.05	-	(0.9603)
Pb	0.12	0.17	0.38	0.15	**0.49**	**0.47**	0.19	0.25	0.30	0.01	-

**Table 3:** Pearson correlation coefficients (*r*) and *p*-values for correlation between *cgMT* expression levels and heavy metals in liver tissue of 155 *C. gariepinus* fish collected over three seasons, conducted separately for each of four sites.

Metal	Chimfunshi		Chililabombwe		Kafue Flats		Kafue Town	
	*r*	*p*	*r*	*p*	*r*	*p*	*r*	*p*
Al	0.20	0.2896	0.06	0.7389	0.10	0.5186	0.15	0.2775
Cr	-	-	−0.19	0.2840	-	-	-	-
Mn	0.29	0.1102	0.09	0.5927	−0.09	0.5530	−0.09	0.5326
Fe	0.05	0.7673	−0.32	0.0608	0.17	0.2675	0.23	0.0892
Co	0.08	0.6564	0.20	0.2429	−0.16	0.2867	0.04	0.7789
Cu	**0.39**	0.0270 *	**0.40**	0.0162 *	**0.37**	0.0135 *	**0.58**	<0.0001 *
Zn	**0.52**	0.0024 *	−0.04	0.8096	**0.45**	0.0022 *	**0.47**	0.0003 *
Se	−0.12	0.5094	**0.57**	0.0003 *	−0.03	0.8677	0.10	0.4710
Cd	−0.34	0.0572	**0.40**	0.0172 *	0.08	0.6277	-	-
Hg	0.22	0.2197	−0.11	0.5482	**0.38**	0.0101 *	**0.35**	0.0097 *
Pb	−0.26	0.1493	0.16	0.3699	0.18	0.2410	-	-

Significant correlations (*p* < 0.05) are marked with * and are highlighted in bold. Entries with no data concern metals that were not detected at the respective site.

**Table 4:** Sequences of the probes and primers used for the real-time quantitative PCR assays.

Gene	Primer/Probe	Sequence
*cgMT*	Forward primer	5′-ACTGCCAGTGCAAATCCTG-3′
	Reverse primer	5′-TCCTGAGGCACACTTACTGC-3′
	Probe	5′-TGCTGCTC-3′
*cgβ-actin*	Forward primer	5′-AGACACCAGGGTGTGATGGT-3′
	Reverse primer	5′-GCTCTGAGCTTCATCACCA-3′
	Probe	5′-GACCCAGA-3′

## Supplementary Materials

Supplementary materials can be found at www.mdpi.com/1422-0067/18/7/1548/s1.

## Abbreviations

AEX	Anion exchange chromatography
BLAST	Basic local alignment search tool
BLASTN	Nucleotide BLAST
BLASTP	Protein BLAST
cDNA	Complementary DNA
CDS	Coding sequence
CgMT	*Clarias gariepinus* metallothionein protein
cgMT	*Clarias gariepinus* metallothionein coding sequence
EDTA	2,2′,2′′,2′′′-(Ethane-1,2-diyldinitrilo)tetraacetic acid
ESI-MS	Electrospray ionsation mass spectrometry
fMet	Formylated methionine
HSD	Honest significant difference
ICP-MS	Inductively-coupled plasma optical emission spectroscopy
ICP-MS	Inductively-coupled plasma mass spectrometry
LB	Lysogeny broth
mRNA	Messenger RNA
MT	Metallothionein
NCBI	National Center for Biotechnology Information
PCR	Polymerase chain reaction
PLI	Pollution load index
qPCR	Quantitative PCR
RI	Potential ecological risk index
RT-PCR	Reverse transcription PCR
SDS-PAGE	Sodium dodecyl sulphate polyacrylamide gel electrophoresis
SEC	Size-exclusion chromatography
