# Supplementary material for: Metallothionein from Wild Populations of the African Catfish Clarias gariepinus: From Sequence, Protein Expression and Metal Binding Properties to Transcriptional Biomarker of Metal Pollution
Source: Int J Mol Sci. 2017 Jul 18;18(7):1548. doi: 10.3390/ijms18071548 (PMC5536036; doi:10.3390/ijms18071548)
Supplement: Supplementary file 1 [file ijms-18-01548-s001.pdf]

Metallothionein from wild populations of the African catfish *Clarias gariepinus*: from sequence, protein expression and metal binding properties to transcriptional biomarker of metal pollution

Supplementary Material

Figure S1: *Clarias gariepinus* MT (*cgMT)* PCR products

Figure S2: cDNA sequences for different PCR products

Table S1: Concentrations of selected metals in Kafue River sediments at four study sites in all three seasons

Figure S3: Metal enrichment in sediments of the Kafue River

Table S2: Method validation for determination of heavy metal content in fish tissues

Table S3: Heavy metal contents (ppm, based on dry weight) in *C. gariepinus* livers

Figure S4: Graphical representation of Cr, Fe, Pb, Se levels in *C. gariepinus* livers

Table S4: Pearson correlation analysis of metals in sediment vs. fish liver samples across all study sites and seasons

Table S5: Pearson correlation analysis of *cgMT* expression levels and heavy metals in livers of *C. gariepinus* across all four study sites and all three seasons

Table S6: Pearson correlation analysis of metals in sediment vs. *cgMT* expression levels across all study sites and seasons

Table S7: PLI and RI values from sediment data

Figure S5: Genomic sequence of *C. gariepinus* MT, with exons and RT-qPCR primers highlighted

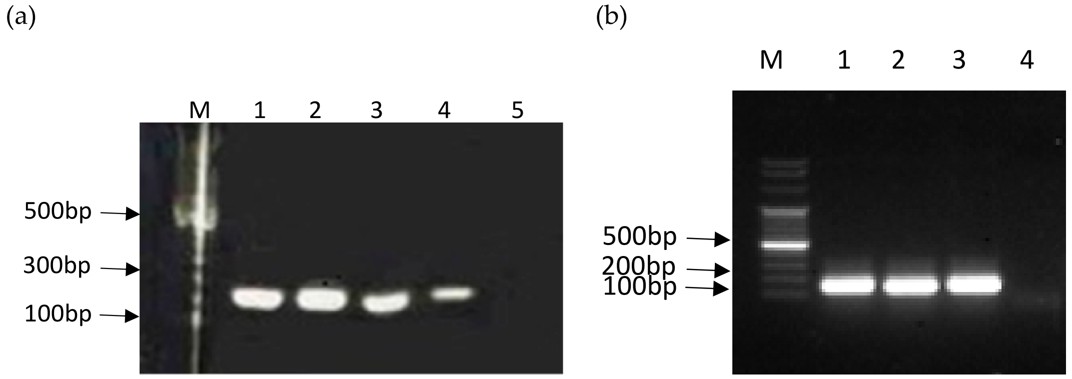

Figure S1. (a) Representative PCR products for *Clarias gariepinus* MT (*cgMT)* derived from using degenerate primers on total cDNA from wild *C. gariepinus* livers. M – DNA ladder (Perfect 100 bp DNA ladder; EURx, Poland), lanes 1-4 – PCR products obtained from four different livers, lane 5 – negative control (without template - cDNA). **(b)** PCR products for *cgMT* coding sequence used for cloning into pET21 vector for expression, containing restriction sites for *NdeI* and *XhoI*. Lane M – DNA ladder (Perfect 100 bp DNA ladder; EURx, Poland), lanes 1-3 – PCR product, lane 4 – negative control (without template - plasmid DNA).

(a)

ATGGACCCCTGCGAGTGTTCAAAGACTGGAACCTGCACgtGCGGTACATCCTGCAAATGCTC
CAACTGCCAGTGCAAATCCTGCAAGAAAAGTTGCTGCTCTTGCTGCCCTTCTAGCTGCAGTA
AGTGTGCCTCAGGATGTGTGTGCAAGGGAGATACCTGTGATTCCAAGTGCTGTCAGTGA

M D P C E C S K T G T C T C G T S C K C S N C Q C K S C K K S C C S C C P S S C S K C A S G C V C K
G D T C D S K C C Q

(b)

ATGGACCCCTGCGAGTGTTCAAAGACTGGAACCTGCACgtGCGGTACATCCTGCAAATGCTC
CAACTGCCAGTGCAAATCCTGCAAGAAAAGTTGCTGCTCTTGCTGCCCTTCTGGCTGCAGTA
AGTGTGCCTCAGGATGTGTGTGCAAGGGAGATACCTGTGATTCCAAGTGCTGTCAGTGA

M D P C E C S K T G T C T C G T S C K C S N C Q C K S C K K S C C S C C P S G C S K C A S G C V C
K G D T C D S K C C Q

(c)

ATGGACCCCTGCGAGTGTTCAAAGACTGGAACCTGCACgtGCGGTACATCCTGCAAATGCTC
CAACTGCCAGTGCAAATCCTGCAAGAAAAGTTGCTGCGCTTGCTGCCCTTCTGGCTGCAGTA
AGTGTGCCTCAGGATGTGTGTGCAAGGGAGATACCTGTGATTCCAAGTGCTGTCAGTGA

M D P C E C S K T G T C T C G T S C K C S N C Q C K S C K K S C C A C C P S G C S K C A S G C V C
K G D T C D S K C C Q

Figure S2. Three cDNA sequences obtained for *cgMT* with two sites of single-nucleotide polymorphism (T/G and A/G) highlighted. The translated sequences are also shown, with amino acid changes (S/A and S/G) highlighted. Sequence **(a)** was the most frequent and was chosen for deposition and cloning for overexpression.

Table S1. Concentrations (mean ± SD, ppm, dry-wt.) of selected metals in Kafue River sediments at four study sites in all three seasons. Data previously published in reference [1]. Chimfunshi is a site unaffected by anthropogenic pollution, Chililabombwe lies within the Copperbelt mining region, and Kafue Flats and Kafue Town are a long distance downstream from the mining area. Also see supplementary Figure S3.

Season	Site		^{27}Al	^{53}Cr	^{55}Mn	^{56}Fe	^{59}Co	^{60}Ni	^{63}Cu	^{66}Zn	^{75}As	^{82}Se	^{111}Cd	^{202}Hg	^{208}Pb
Warm-rainy	Chimfunshi	Mean	29204	30.3	128	10224	13.9	16.2	73.5	16.8	1.58	0.38	0.03	0.22	7.58
Warm-rainy		SD	1334	1.22	3.7	659	0.17	0.3	2.84	0.98	0.11	0.66	0.05	0.19	0.72
Warm-rainy	Chililabombwe	Mean	46925	33.3	1537	22433	394	21	10671	58.8	2.54	2.82	0.02	0.2	19.8
Warm-rainy		SD	1316	1.68	106	527	19.3	1.38	477	5.57	0.25	0.61	0.03	0.03	0.88
Warm-rainy	Kafue Flats	Mean	62796	51.5	81.8	21600	11.4	27.4	29.3	22.9	1.64	0.7	ND	0.1	7.54
Warm-rainy		SD	3480	2.6	6.3	1186	0.67	1.54	1.81	1.71	0.15	0.03	ND	0.05	0.83
Warm-rainy	Kafue Town	Mean	19482	18	53.4	9371	5.08	8.84	33.9	34.4	2.01	0.25	0.01	0.1	17.7
Warm-rainy		SD	1991	0.83	1.13	649	0.25	0.18	1.65	4.6	0.58	0.43	0.02	0.03	1.34
Dry-cold	Chimfunshi	Mean	21487	24.7	83.8	9384	18.6	13.8	78.8	26.6	1.62	0.27	0.29	0.09	5.47
Dry-cold		SD	3143	1.02	2.56	388	0.62	0.56	2.64	2.24	0.12	0.47	0.13	0.03	1
Dry-cold	Chililabombwe	Mean	28249	27.9	498	17051	248	18.8	5660	57.5	1.87	0.77	0.06	0.07	11.88
Dry-cold		SD	672	0.79	21.7	2061	5.71	0.6	442	1.79	0.13	0.75	0.07	0.02	1.27
Dry-cold	Kafue Flats	Mean	60536	48.9	108	25515	12.3	23.7	33.7	25.9	1.72	ND	ND	0.05	7.84
Dry-cold		SD	1168	1.71	16.6	3627	0.66	1.02	3.12	2.46	0.01	ND	ND	0.01	1.86
Dry-cold	Kafue Town	Mean	27116	33.4	64.1	14649	6.45	17.2	17.8	9.75	1.35	ND	0.16	0.04	4.42
Dry-cold		SD	2844	3.05	9.15	3037	0.9	1.84	3.26	3.05	0.03	ND	0.1	0.01	1.91
Dry-hot	Chimfunshi	Mean	30560	36	49.2	11483	12.4	22.1	39.9	16	0.94	0.001	0.3	0.13	6.17
Dry-hot		SD	6986	13.8	17.8	4094	4.12	7.36	13.2	8.35	0.32	0.0003	0.17	0.09	3.65
Dry-hot	Chililabombwe	Mean	16753	20.6	603	16324	338	16.7	12037	44.7	1.89	0.76	0.02	0.14	14.6
Dry-hot		SD	3202	5.49	143	4015	48	3.1	1810	14.4	0.36	1.32	0.04	0.08	4.88
Dry-hot	Kafue Flats	Mean	53101	47.4	141	25132	12.9	23.2	40.9	23.9	1.48	6.6	0.01	0.11	7.64
Dry-hot		SD	2935	11.4	45.3	7687	2.84	4.9	10.4	9.69	0.38	0.2	0.01	0.09	4.12
Dry-hot	Kafue Town	Mean	47741	63.4	141	31407	11.6	69.8	23.3	14.5	1.37	4.49	ND	0.09	4.98
Dry-hot		SD	9836	19.7	44.5	10734	3.1	23.7	6.98	7.35	0.28	3.9	ND	0.09	3.28

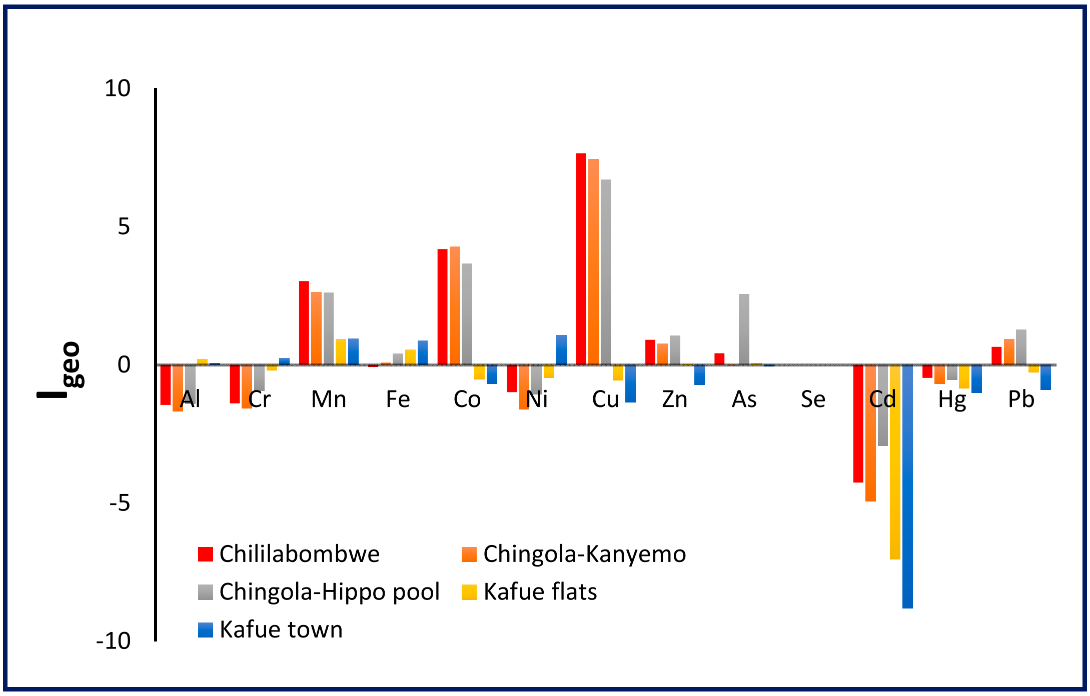

Figure S3. Relative enrichment of different metals in sediments at 5 sites downstream from Chimfunshi (reference site), for the hot season (data taken from ref. [1]. The same trends were observed in the other two seasons. The I_{geo} value describes the degree of anthropogenic enrichment of heavy metals in sediment and was calculated according to ref. [2]: $I_{geo} = \log_2 (C_c/1.5C_b)$, where C_c is the measured concentration of each heavy metal in sediment at a contaminated site and C_b is the background concentration of each heavy metal as determined for the uncontaminated reference site. A high enrichment of Cu, Co, and Mn at the three sites in/downstream of the Copperbelt mining area (Chililabombwe, Chingola Kanyemo, Chingola Hippo pool) is clearly evident. Smaller enrichments of Zn and Pb are also observed, whereas Cr, Cd, Ni and Hg are less abundant downstream compared to upstream. Fe is only very slightly enriched at the three sits furthest downstream, and As levels are increased in Chingola Hippo pool. Detection levels for Se were too low at most sites, so no data are given.

References:

1. M'kandawire, E.; Choongo, K.; Yabe, J.; Mwase, M.; Saasa, N.; Nakayama, S.M.M.; Bortey-Sam, N.; Blindauer, C.A. Sediment metal contamination in the Kafue River of Zambia and ecological risk assessment. *Bull. Environ. Contam. Toxicol.* **2017**, *in press*. doi:10.1007/s00128-017-2089-3.
2. Loska, K., Wiechuła, D., Korus, I. Metal contamination of farming soils affected by industry. *Environ. Int.* **2004**, *30*, 159-165.

Table S2. Measured concentrations of heavy metals (ppm, based on dry weight) in fish muscle certified reference material (ERM® - BB422) in comparison with certified values for this CRM.

Element/ isotope	Measured mean value (ppm)	Measured standard deviation (ppm)	CRM certified value (ppm)	CRM uncertainty value (ppm)	Percent recoveries (%)
^{27}Al	ND	ND	-	-	-
^{53}Cr	ND	ND	-	-	-
^{55}Mn	0.36	0.02	0.368	0.028	98.3
^{56}Fe	9.20	0.40	9.40	1.4	98.0
^{59}Co	ND	ND	-	-	-
^{60}Ni	ND	ND	-	-	-
^{63}Cu	1.41	0.05	1.67	0.16	84.2
^{66}Zn	20.9	1.01	16.0	1.1	129.4
^{75}As	12.8	0.11	12.7	0.7	101.0
^{82}Se	1.17	0.11	1.33	0.13	88.3
^{111}Cd	0.007	0.0002	0.0075	0.0018	95.1
^{202}Hg	0.73	0.07	0.601	0.030	121.3
^{208}Pb	ND	ND	-	-	-

ND=Not detected

Table S3: Concentrations (mean ± SD) of heavy metals (ppm, based on dry weight) in *C. gariepinus* livers. Statistical analysis by site in each season is included. Levels not connected by the same letter (a-d) are significantly different ($p < 0.05$) among sites.

Season	Site	N		^{27}Al	^{53}Cr	^{55}Mn	^{56}Fe	^{59}Co	^{60}Ni	^{63}Cu	^{66}Zn	^{75}As	^{82}Se	^{111}Cd	^{202}Hg	^{208}Pb
Warm-rainy	Chimfunshi	10	Mean	18.9a	ND	6.18a	3227b	0.88b	ND	58.1b	144b	ND	33.3bc	0.81a	0.21a	ND
			SD	7.05	ND	2.65	1045	0.56	ND	28.0	46.8	ND	26.9	0.96	0.11	ND
Warm-rainy	Chililabombwe	10	Mean	12.4a	0.34a	6.20a	9091a	2.19a	0.02	531a	247a	ND	51.7abc	0.45a	0.22a	ND
			SD	6.02	0.54	0.90	4180	1.00	0.07	357	77.6	ND	27.9	0.58	0.12	ND
Warm-rainy	Kafue Flats	13	Mean	13.2a	ND	5.06ab	5970ab	0.64b	ND	97.2b	174b	ND	103ab	ND	0.13a	ND
			SD	9.61	ND	0.90	3483	0.61	ND	53.1	70.5	ND	124	ND	0.04	ND
Warm-rainy	Kafue Town	13	Mean	12.3a	0.02b	4.08b	4273b	0.44b	ND	61.9b	155b	ND	24.0c	ND	0.17a	ND
			SD	8.42	0.06	0.78	2316	0.20	ND	28.7	39.1	ND	5.2	ND	0.05	ND
Dry-cold	Chimfunshi	11	Mean	1.17c	0.02c	3.85d	4400bc	0.90b	ND	58.8d	136b	ND	101a	1.41a	0.17a	0.09a
			SD	1.16	0.04	0.54	2262	0.31	ND	20.0	28.8	ND	98.6	1.00	0.03	0.03
Dry-cold	Chililabombwe	12	Mean	14.0ab	0.14b	6.44a	9566a	1.85a	ND	173a	248a	ND	53.0a	0.16b	0.25a	0.12a
			SD	15.1	0.27	2.02	3470	0.56	ND	26.1	75.4	ND	16.3	0.19	0.11	0.05
Dry-cold	Kafue Flats	16	Mean	28.9a	0.01c	4.38bcd	6456ab	0.73bc	ND	90.2b	189ab	ND	58.4a	0.12b	0.25a	0.08a
			SD	41.7	0.04	1.74	3058	0.32	ND	22.4	43.3	ND	58.8	0.18	0.12	0.04
Dry-cold	Kafue Town	14	Mean	9.07b	0.35a	5.45ab	3717c	0.52cd	ND	78.7bc	200ab	ND	97.9a	0.01b	0.25a	ND
			SD	6.00	1.14	1.26	2871	0.13	ND	17.2	86.4	ND	131	0.02	0.06	ND
Dry-hot	Chimfunshi	10	Mean	5.79bc	0.004b	4.76a	5756ab	0.92bc	ND	71.7b	160ab	ND	48.3bc	1.81a	0.24a	0.15b
			SD	2.65	0.01	1.00	1847	0.61	ND	26.8	31.3	ND	24.3	2.37	0.10	0.12
Dry-hot	Chililabombwe	17	Mean	8.45bc	0.08a	6.08a	6230a	3.10a	ND	473a	204a	ND	126a	1.61ab	0.22a	0.49a
			SD	9.77	0.24	2.70	1353	1.73	ND	564	56.1	ND	66.4	1.65	0.10	0.94
Dry-hot	Kafue Flats	18	Mean	19.4a	ND	5.01a	7307a	0.42bc	ND	78.1b	166ab	ND	52.2b	0.04c	0.22a	0.10b
			SD	8.90	ND	1.07	4349	0.11	ND	37.3	39.5	ND	19.5	0.02	0.07	0.03
Dry-hot	Kafue Town	11	Mean	8.69ab	ND	4.54a	2929b	0.39c	ND	61.5b	141b	ND	27.8c	0.09bc	0.29a	0.10b
			SD	4.07	ND	0.98	1920	0.07	ND	26.3	35.1	ND	11.3	0.07	0.10	0.02

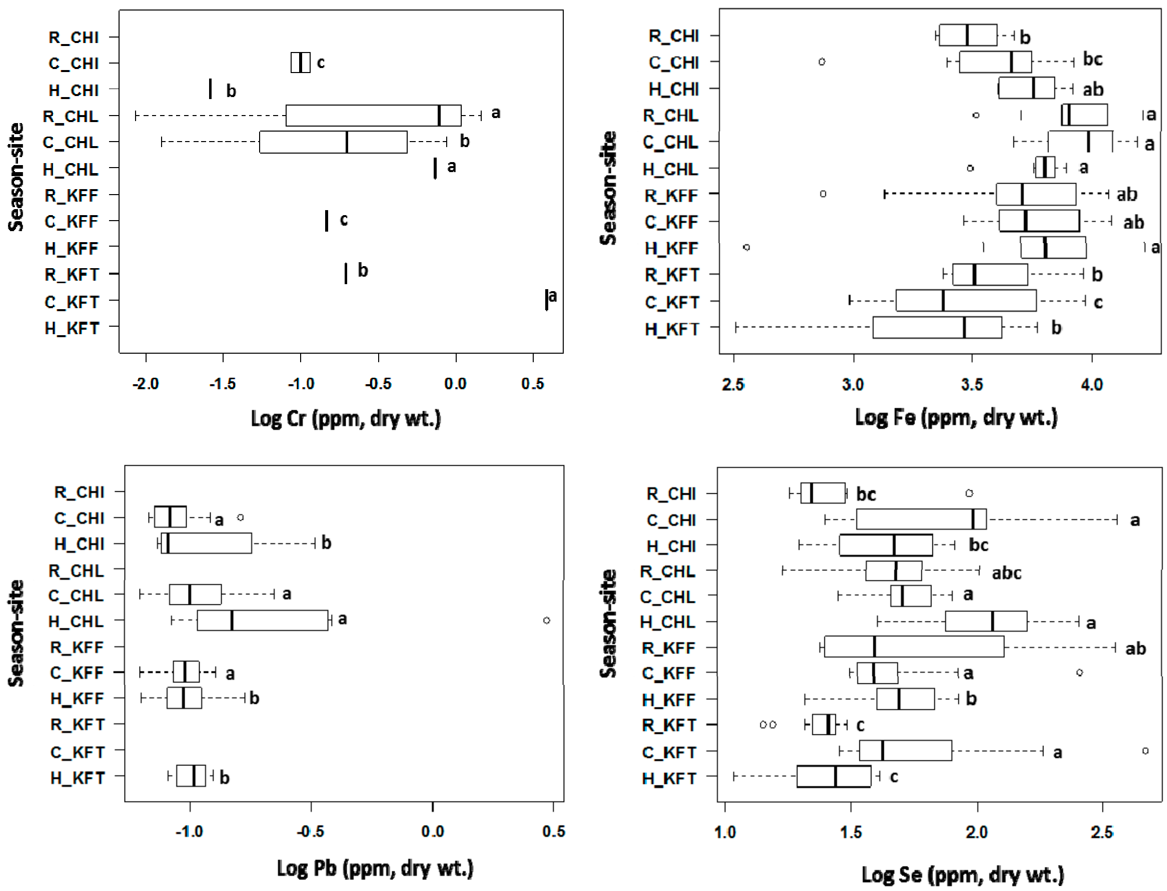

Figure S4. Levels of Cr, Fe, Pb and Se in liver tissue of *C. gariepinus* collected from 4 sample sites (CHI = Chimfunshi, CHL = Chililabombwe, KFF = Kafue Flats, KFT = Kafue Town) during three distinct seasons (R = warm-rainy season, C = dry-cold season, H = dry-hot season). One way ANOVA followed by a post-hoc Tukey's (HSD) test was applied to assess for spatial and seasonal differences in heavy metal levels. Levels for individual metals not connected by the same letter ('a', 'b' or c') are significantly different ($p < 0.05$) between sites, as analysed for each individual season.

Table S4. Pearson correlation analysis of metals in sediment [81] and fish liver samples across all study sites and seasons

Metal	correlation coefficient	p-value
Al	**0.40**	**<0.00001***
Cr	-0.14	0.6049
Mn	**0.38**	**<0.00001***
Fe	-0.06	0.486
Co	**0.78**	**<0.000001***
Cu	**0.73**	**<0.000001***
Zn	0.33	0.0003*
Se	0.04	0.7447
Cd	**0.40**	**<0.00001***
Hg	-0.17	0.05665
Pb	0.34	0.00028*

* indicates significant correlations ($p < 0.05$); the most significant correlations are highlighted in bold.

Table S5. Pearson correlations and p-values between *cgMT* expression levels and heavy metals in liver tissue of *C. gariepinus* across all four study sites over all three seasons.

Metal	correlation coefficient with *cgMT* expression	p-value
Al	0.07	0.4203
Cr	0.03	0.7616
Mn	0.25	0.0018*
Fe	0.22	0.0061*
**Co**	**0.54**	**<0.00001***
**Cu**	**0.61**	**<0.00001***
**Zn**	**0.44**	**<0.00001***
Se	0.23	0.0043*
Cd	0.20	0.0128*
Hg	0.14	0.0955
Pb	0.25	0.0020*

* indicates significant correlations (p < 0.05); the most significant correlations are highlighted in bold.

Table S6. Pearson correlation coefficients (r) and p-values for correlation between *cgMT* expression levels in livers of 155 *C. gariepinus* fish collected at all four sites over three seasons and heavy metals in sediment samples collected at the same four sites and in the same three seasons.

**Metal**	**r**	**p**
Al	–1.2157	0.0084
As	0.1361	0.0988
Cd	–0.1883	0.0478
**Co**	**0.6501**	**<0.00001***
Cr	–0.1789	0.0296
**Cu**	**0.6696**	**<0.00001***
Fe	0.0861	0.2982
Hg	0.1797	0.0591
**Mn**	**0.4422**	**<0.00001***
Ni	–0.0644	0.4369
**Pb**	**0.5019**	**<0.00001***
Se	0.1752	0.0659
**Zn**	**0.6403**	**<0.00001***

Table S7. Pollution load index (PLI) and potential ecological risk index (RI) determined from metal concentrations in Kafue River sediments. Data taken from [81]; the PLI values are based on all studied metals; the RI values incorporate Cr, Ni, Cu, Zn, As, Cd, Hg and Pb.

Season	Site	PLI	RI
Warm-rainy	Chililabombwe	3.58	823
Warm-rainy	Kafue flats	1.09	49
Warm-rainy	Kafue town	0.71	66
Dry-cold	Chililabombwe	2.32	431
Dry-cold	Kafue flats	1.17	55
Dry-cold	Kafue town	0.72	59
Dry-hot	Chililabombwe	2.25	1595
Dry-hot	Kafue flats	0.94	70
Dry-hot	Kafue town	0.84	72

TAATGGACCCCTGCGAGTGTTCAAAGAGTGAGTAGCTGTAGAGTTTTATGTTAATATTGTTTATTTCTCTTCACC
TGTGTCCTGACTTGGTAAATAACACTTGTTAACGGTACTGTGTTCTCTCTCTGCTTTCTGCAGCTGGAACCTGCA
CGTGCGGTACATCCTGCAAATGCTCCAACTGCCAGTGCAAATCCTGCAAGAAAAGTAAGCTCTTTTAAAATCTCC
TGAATAATGGAAATCTTGCTTTAATTCCTGACTCACCAGTACATTTCCTTTCCCCAGGTTGCTGCTCTTGCTGCC
CTTCTAGCTGCAGTAAGTGTGCCTCAGGATGTGTGTGCAAGGGAGATACCTGTGATTCCAAGTGCTGTCAGTGAA
C

Figure S5. Genomic DNA sequence of *Clarias gariepinus* MT. Exons are underlined, and primers used for RT-qPCR are highlighted in green. Start and stop codons are highlighted in yellow.
